# Monoamine Oxidase Inhibitors: A Review of Their Anti-Inflammatory Therapeutic Potential and Mechanisms of Action

**DOI:** 10.3389/fphar.2021.676239

**Published:** 2021-04-30

**Authors:** Mahyar Ostadkarampour, Edward E. Putnins

**Affiliations:** Department of Oral Biological and Medical Sciences, Faculty of Dentistry, The University of British Columbia, Vancouver, BC, Canada

**Keywords:** monoamine oxidases, inhibitors, inflammation, cytokines, osteoclastogenesis, catecholamines

## Abstract

Chronic inflammatory diseases are debilitating, affect patients’ quality of life, and are a significant financial burden on health care. Inflammation is regulated by pro-inflammatory cytokines and chemokines that are expressed by immune and non-immune cells, and their expression is highly controlled, both spatially and temporally. Their dysregulation is a hallmark of chronic inflammatory and autoimmune diseases. Significant evidence supports that monoamine oxidase (MAO) inhibitor drugs have anti-inflammatory effects. MAO inhibitors are principally prescribed for the management of a variety of central nervous system (CNS)-associated diseases such as depression, Alzheimer’s, and Parkinson’s; however, they also have anti-inflammatory effects in the CNS and a variety of non-CNS tissues. To bolster support for their development as anti-inflammatories, it is critical to elucidate their mechanism(s) of action. MAO inhibitors decrease the generation of end products such as hydrogen peroxide, aldehyde, and ammonium. They also inhibit biogenic amine degradation, and this increases cellular and pericellular catecholamines in a variety of immune and some non-immune cells. This decrease in end product metabolites and increase in catecholamines can play a significant role in the anti-inflammatory effects of MAO inhibitors. This review examines MAO inhibitor effects on inflammation in a variety of *in vitro* and *in vivo* CNS and non-CNS disease models, as well as their anti-inflammatory mechanism(s) of action.

## Introduction

Inflammation is orchestrated by local and recruited immune cells in response to pathogens, damaged tissue, toxic compounds, and irritants (Chen et al., 2018). Cytokines and chemokines as a complex network act as molecular messengers that signal through a myriad of receptors to regulate inflammation (Borish and Steinke, 2003; Rea et al., 2018). Collectively, they are secreted by immune and non-immune cells, and play a role in acute and chronic inflammation and autoimmune diseases (Gabay and Kushner, 1999; Borish and Steinke, 2003; Balkwill, 2009; Tisoncik et al., 2012; Tanaka et al., 2014; Hunter and Jones, 2015; Dinarello, 2018). Chronic inflammation affects a variety of soft tissues and organs such as the gastrointestinal track, heart, and brain (Chung et al., 2009; Cao et al., 2015; Straub and Schradin, 2016). In addition, inflammation can disrupt bone homeostasis, leading to osteoclast-mediated bone loss (Redlich and Smolen, 2012). Anti-inflammatories that target cytokines and chemokines have been developed for the treatment of soft and hard tissue chronic inflammatory and autoimmune diseases (Tisoncik et al., 2012; Campbell et al., 2013; Tanaka et al., 2014; Dinarello, 2018). A growing body of evidence demonstrates in a number of *in vitro* and *in vivo* disease models that MAO-A, MAO-B, and MAO-A/B inhibitors reduce mediators of inflammation and tissue destruction.

Monoamine oxidases (MAOs) are mammalian flavoenzymes (EC 1.4.3.4) that catalyze the oxidative deamination of biogenic and dietary amines, monoamine hormones, and neurotransmitters such as serotonin, dopamine, norepinephrine, and epinephrine, as well as a number of trace amines, such as tyramine, tryptamine, and 2-phenylethylamine (Bortolato and Shih, 2011; Ramsay and Albreht, 2018; Tipton, 2018). Two isoforms of MAO (MAO-A and MAO-B) have been identified, but they differ in substrate specificities, inhibitor affinity, relative expression, and tissue localization (Tipton, 2018). MAO-A has high affinity for serotonin and to a lesser degree norepinephrine. MAO-B more effectively metabolizes phenylethylamine and benzylamine. Epinephrine, dopamine, tryptamine, and tyramine are metabolized to varying degrees by both MAO-A and MAO-B. MAOs are encoded by two distinct genes that are located on the X chromosome (Xp11.23); both have identical exon–intron organization and share 70 percent amino acid identity (Grimsby et al., 1991; Gaweska and Fitzpatrick, 2011). Both enzymes are dimeric in their membrane-bound form (Son et al., 2008; Binda et al., 2011). For rat liver, MAO-A and MAO-B are anchored to the mitochondrial membrane through a hydrophobic C-terminal α-helix and are oriented to the cytosolic face or intermembrane space of the mitochondrial outer membrane, respectively (Wang and Edmondson, 2011; Edmondson and Binda, 2018; Iacovino et al., 2018). In any case, this orientation may vary between tissues and species.

Deletion of the MAO-A and MAO-B genes are not lethal but Norrie disease patients with MAO-A and MAO-B gene deletions show severe intellectual disability, growth failure, alteration of sleep patterns, autistic-like symptoms, and bilateral congenital blindness (Bortolato et al., 2018; Rodriguez-Munoz et al., 2018). MAO-A gene mutations were found in patients with Brunner syndrome. These males showed mild intellectual disability with aggressive and, at times, violent behavior (Brunner et al., 1993). MAO-A knockout (KO) mice demonstrate elevated aggressiveness and autistic-like behavior, and elevation of serotonin (200%), norepinephrine (130%), and dopamine (110%). In contrast, MAO-B KO mice exhibit lower anxiety-like responses and shorter latency to partake in risk-taking behavior, explore new objects, and have significantly elevated levels of phenylethylamine (700%) (Bortolato et al., 2008; Bortolato and Shih, 2011). MAO-A/B KO mice expressed developmental changes, behavioral abnormalities, and significantly elevated levels of all amines that far exceeded what was found in single knockout animals. Observed changes in these animals may reflect exposure to high levels of monoamines during developmental stages (Bortolato et al., 2008; Bortolato et al., 2009; Bortolato and Shih, 2011).

## Non-CNS Expression of MAO-A and MAO-B

Historically, most studies on the function and localization of MAO enzymes have focused on the distribution and role of MAOs in the central nervous system, although widespread expression of MAO mRNA is found (Sivasubramaniam et al., 2003). MAO enzymes are widely expressed in different organs such as the heart, lungs, intestine, kidney, and liver but differences in enzyme isotype do exist (Rodriguez et al., 2001; Sivasubramaniam et al., 2003). MAO-A is preferentially expressed in the gastrointestinal track and found in moderately higher levels in human heart. MAO-B is preferentially expressed in kidney, platelets, granulocytes, and lymphocytes with a relatively equal distribution in the lungs, spleen, and liver (Thorpe et al., 1987; Balsa et al., 1989; Pizzinat et al., 1999; Rodriguez et al., 2001; Sivasubramaniam et al., 2002; Billett, 2004; Tripathi et al., 2018). MAO-B was highly upregulated in lipopolysaccharide (LPS)-induced periodontal disease (Ekuni et al., 2009). MAO-A was one of the five most significantly upregulated genes in interleukin-4-induced alternate activation of monocytes/macrophages (Chaitidis et al., 2004; Chaitidis et al., 2005; Gordon and Martinez, 2010; Cathcart and Bhattacharjee, 2014).

## Monoamine Oxidase Enzyme Activity

**FIGURE 1:**
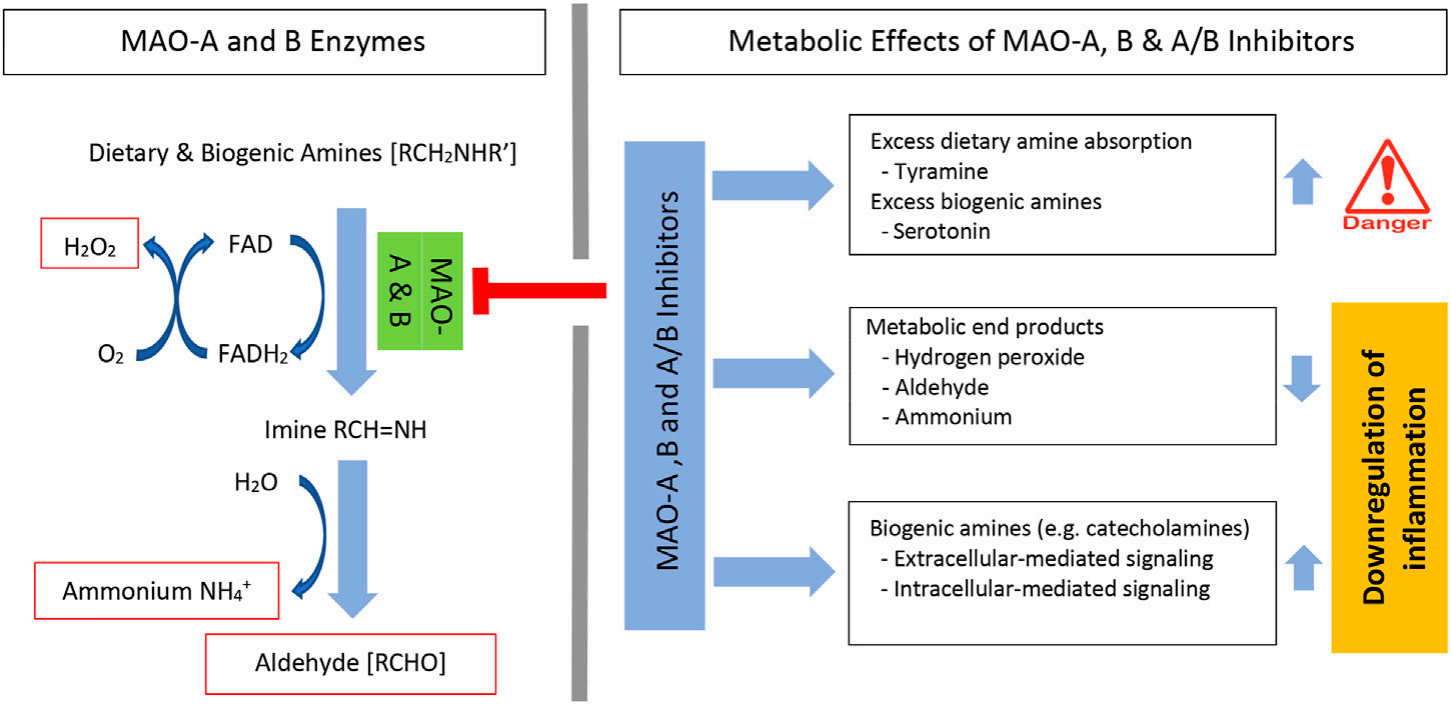
MAO oxidative deamination of amines and MAO inhibitor metabolic effects. Systemic levels of dietary and biogenic amines are regulated by MAO-A and MAO-B enzymes. Enzyme activity forms three specific metabolic end products. These include hydrogen peroxide (H_2_O_2_), aldehyde, and ammonium; each can affect inflammation (left). Irreversible MAO inhibitors can induce significant absorption of dietary amines (e.g., tyramine) and increase biogenic amine levels (e.g., serotonin) (right). MAO inhibitors decrease metabolic end products and increase catecholamines. Both the decrease in metabolic end products and the increase in catecholamines regulate inflammation. FAD, flavin adenine dinucleotide; FADH_2_, dihydroflavin adenine dinucleotide.

MAO enzyme activity requires flavin adenine dinucleotide (FAD), which is covalently bound to a cysteine residue on the enzymes. FAD is reduced to its hydroquinone (FADH_2_) while the amine is reduced to the corresponding imine. This catalytic process has not been fully resolved, however, using semiempirical quantum mechanics/molecular mechanics (QM/MM) simulations, it was shown that hydride transfer from the substrate onto the flavin moiety was rate limiting (Repic et al., 2014; Prah et al., 2020). Once dissociated from the enzyme, the imine is spontaneously hydrolyzed with generation of aldehyde [RCHO] and ammonium [NH_4_^+^]. Subsequently, the FADH_2_ is reoxidized to FAD and this results in the formation of another major metabolic end product, hydrogen peroxide [H_2_O_2_] (Bortolato and Shih, 2011; Gaweska and Fitzpatrick, 2011; Ramsay and Albreht, 2018) (Figure 1-left). Inhibition of MAO enzymes decreases these metabolic end products and increases the availability of dietary and biogenic amines. This increase in amines has clinical therapeutic effects, but also has some negative side effects (Figure 1-top right).

## Pharmacological Control of MAO Enzymes: MAO Inhibitors

MAO-A and MAO-B inhibitor drugs are primarily prescribed for the control of emotional behavior (e.g. depression and anxiety disorders) and neurodegenerative diseases (e.g. Parkinson’s disease, Alzheimer’s, and possibly amyotrophic lateral sclerosis and Huntington’s diseases) (Bortolato et al., 2008; Dlugos et al., 2009; Bortolato and Shih, 2011; Youdim et al., 2006; Al-Nuaimi et al., 2012; Tripathi et al., 2018). MAO inhibitors bind to MAO enzymes in either a reversible or an irreversible manner (Ramsay and Albreht, 2018; Tipton, 2018). Irreversible inhibitors bind to the enzyme by forming a covalent enzyme-inhibitor bond, typically with the enzyme-bound FAD. This is time dependent and not reversed by dialysis. Using QM/MM simulations, the rate-limiting step found for rasagiline and selegiline (irreversible MAO-B inhibitors)-mediated inhibition was hydride anion transfer from the inhibitors onto the FAD co-factor (Tandaric et al., 2020). In contrast, reversible inhibitors inactivate the enzyme through weak interactions such as hydrogen bonds (Edmondson and Binda, 2018). The efficacy of the inhibitor binding into the unique substrate binding sites of MAO-A and MAO-B ultimately determines a drug’s relative selectivity at inhibiting MAO-A, MAO-B, or both. The substrate binding sites of both MAO-A and MAO-B are mainly hydrophobic except for a conserved lysine that interacts with a water molecule. A list of the MAO inhibitor drugs discussed in this article with anti-inflammatory effects and their relative selectivity and reversibility is summarized (Table 1).

**TABLE 1:** MAO reversible and irreversible inhibitors with described anti-inflammatory effects.

Drug	MAO selectivity	Clinical indication(s)
Irreversible MAO inhibitors		
Clorgyline	A	Depression
l-Deprenyl (selegiline)	B	Parkinson’s
Iproniazid	A and B	Depression
Isocarboxazid	A and B	Depression
Ladostigil	A and B (brain selective)	Depression, Parkinson’s, and Alzheimer’s
Nialamide	A and B	Depression
Phenelzine	A and B	Depression
Pargyline	B	Hypertension (discontinued)
Rasagiline	B	Parkinson’s
Tranylcypromine	A and B	Depression
Reversible MAO inhibitors		
Bifemelane	A and B	Antidepressant, depression, and dementia
Moclobemide	A	Depression

The first MAO inhibitor was an irreversible and non-selective MAO-A and MAO-B inhibitor, iproniazid (Pletscher, 1991). This hydrazine-based compound was initially developed to treat tuberculosis but was a more effective antidepressant. This spurred the development of other non-selective irreversible inhibitors, but the side effects of hepatotoxicity and hypertensive crisis limited their use (Tipton, 2018). Subsequent non-hydrazine compounds showed reduced liver toxicity; however, hypertensive crisis was a significant side effect that resulted in some patient deaths (Youdim et al., 2006). This occurred because the use of non-selective irreversible MAO inhibitors resulted in significant dietary tyramine absorption (Figure 1-top right). Tyramine is normally metabolized by MAO in the gut, with MAO-A in the gut accounting for 70% of the tyramine degradation and additional metabolization in the liver (50% MAO-A and 50% MAO-B) (Hasan et al., 1988; Youdim et al., 2006). Failure to metabolize tyramine results in its absorption and uptake by peripheral adrenergic neurons, where it displaces norepinephrine and induces a hypertensive event (Blackwell, 1963). This became known as the “cheese effect” because documented fatalities were associated with cheese intake (Gillman, 2018).

Tyramine-induced hypertensive crisis was associated with the use of non-selective MAO inhibitors and irreversible MAO-A inhibitors. MAO-B selective inhibitors can be of concern at high doses when selectivity is lost. Reversible MAO-A inhibitors do not have this effect because the dose to achieve their antidepressant effect is such that dietary tyramine can displace the bound inhibitor and is therefore metabolized (Anderson et al., 1993).

A second major side effect from the use of MAO inhibitors occurs due to biogenic amine excess and its negative physiologic effects (Figure 1-top right). Serotonin syndrome (serotonin toxicity) occurs due to excess serotonin in brain synapses. Cases requiring hospitalization are rare and mild cases are typically not fatal. Patients present with a combination of neuromuscular, autonomic, and mental status symptoms that range from mild symptoms of nervousness, nausea, and tremor to more severe symptoms, such as a fever >38.5 °C, confusion, sustained clonus rigidity, and death. Most cases occur due to the use of two drugs that in different ways increase serotonin levels. These include serotonin-elevating drugs (e.g., monoamine oxidase inhibitors), selective serotonin reuptake inhibitors (SSRIs), and serotonin releasers such as amphetamine and the illicit drug, ecstasy. Non-selective and irreversible MAO-A and MAO-B inhibitors, selective and irreversible MAO-B inhibitors, and selective and reversible MAO-A inhibitors have been associated with serotonin syndrome (Foong et al., 2018). Regardless of some of these drug restrictions, MAO inhibitors have proven to be highly effective for the management of a variety of CNS-associated diseases. Moreover, a growing body of evidence demonstrates that a wide variety of MAO inhibitors also exert anti-inflammatory effects in various tissues.

## MAO Inhibitors: Regulation of Cytokine and Chemokine Expression

Acute and chronic inflammation is orchestrated and driven by cytokines and chemokines expressed by local cells and recruited immune cells. Some of the key players of the pro-inflammatory cytokine network include tumor necrosis factor-α (TNF-α), interleukin-1β (IL-1β), and interleukin-6 (IL-6) (Gabay and Kushner, 1999; Borish and Steinke, 2003; Balkwill, 2009; Tisoncik et al., 2012; Tanaka et al., 2014; Hunter and Jones, 2015; Dinarello, 2018). Interleukin-8 (CXCL8) is the prototypical member of the CXC chemokine family and serves as a potent chemoattractant for neutrophils in humans. IL-8 plays a critical role in cell recruitment during acute infections and is overexpressed in a number of chronic inflammatory diseases (Palomino and Marti, 2015). Regulating levels of these mediators of inflammation is an established approach for managing chronic inflammatory diseases. MAO-A, MAO-B, and MAO-A/B inhibitors have been shown to significantly change cytokine and chemokine expression in a number of cell culture and disease models such as depression, Parkinson’s, ischemia/reperfusion tissue injury, periodontal disease, and smoke-induced lung injury (Table 2).

**TABLE 2:** MAO inhibitor effects on inflammatory cytokine and chemokine expression.

Drug	Clinical use	Cell population	Stimuli	Cytokines and chemokines	Protein change %^a^	mRNA change %^a^	References
**MAO-A Inhibitors**							
Moclobemide	ADP	Rat glial cell	LPS	TNF-α		60↓	Bielecka et al. (2010)
				IL-1β		52 ↓	
Moclobemide	ADP	Human whole blood	LPS + PHA	IL-10	27–33 ↑		Lin et al. (2000)
			Unstimulated	IL-8	21–42 ↓		
			Unstimulated	TNF-α	41–67 ↓		
Clorgyline	ADP	Mouse bone stromal cells	Tumor cell injection	IL-6		40 ↓	Wu et al. (2017)
**MAO-B Inhibitors**							
Pargyline	ADP	Mouse renal tissue	I/R and cyclosporin	TNF-α		50 ↓	Chaaya et al. (2011)
				IL-1β		45 ↓	
L-deprenyl	PD	Rat periodontal epithelial cells	LPS	TNF-α	54 ↓		Ekuni et al. (2009)
L-deprenyl	PD	Human bronchial epithelial cells	CSM	IL-8	18–60 ↓		Cui et al. (2017)
L-deprenyl	PD	Rat BAL	CS	CINC-1	24 ↓		Cui et al. (2020)
				MCP-1	33 ↓		
				IL-6	50 ↓		
				IL-10	68 ↑		
Rasagiline	PD	Rat brain homogenate	Rotenone-induced PD	TNF-α		65 ↓	Fernandez et al. (2011)
Rasagiline	PD	Murine N9 microglia cells	DJ-1 deficiency DV	IL-6	30 ↓		Trudler et al. (2014)
				IL-1β	29 ↓		
Rasagiline	PD	Mouse bone marrow-derived macrophages	LPS/ATP	IL-1β	54 ↓	Reduced to control	Sanchez-Rodriguez et al. (2020)
**MAO-A and B Inhibitors **							
Phenelzine	ADP	Rat periodontal epithelial cells	LPS	TNF-α	70 ↓		Ekuni et al. (2009)
Phenelzine	ADP	Mouse primary and microglial cell line	LPS	TNF-α	67–83 ↑		Chung et al. (2012)
				IL-6	33–43 ↑		
Tranylcypromine	ADP	Rat brain different region	LPS	IL-1β	85–90 ↓		Tomaz et al. (2020)
				IL-6	75–90 ↓		
				TNF-α	35–52 ↓		
				IFN-γ	80–94 ↓		
Nialamide	ADP	Mouse brain cortex cells	–	TNF-α	55 ↓		Liu et al. (2020)
Ladostigil	ADP, PD, AD	Rat microglial Cells	LPS	TNF-α	45 ↓	30 ↓	Panarsky et al. (2012)
				IL-1β		35 ↓	
		Rat partial cortex	–	TNF-α		53 ↓	
				IL-1β		28 ↓	
				IL-6		47 ↓	

AD: Alzheimer’s disease; ADP: antidepressant; ATP: adenosine triphosphate; BAL: bronchoalveolar lavage; CINC-1: cytokine-induced neutrophil chemoattractant; CS: cigarette smoke; CSM: cigarette smoking medium; DV: dopamine vulnerability; I/R: ischemia/reperfusion; LPS: lipopolysaccharide; MAO: monoamine oxidase; MCP-1: monocyte chemoattractant protein 1; PD: Parkinson’s disease; PHA: phytohemagglutinin

a estimated change

A historical case report described a patient who showed a rapid improvement in her Crohn’s disease when prescribed phenelzine (MAO-A/B irreversible inhibitor) for associated depression. Anxiety and depression are risk factors in inflammatory bowel disease and the symptoms are more common during periods of active disease (Graff et al., 2009). The patient’s Crohn’s disease remained stable; however, 6 weeks after stopping the phenelzine, she was readmitted to hospital due to reactivation of her Crohn’s (Kast, 1998). This early clinical report eluded to a potential MAO inhibitor anti-inflammatory effect. In an LPS-induced depression rat model, tranylcypromine (irreversible MAO-A/B inhibitor) decreased the LPS-induced expression of IL-1β, IL-6, TNF-α, and interferon-γ (IFN-γ) in regions of the brain (Tomaz et al., 2020). Tranylcypromine did not alter LPS-mediated NF-κB signaling in the hippocampal region of rat brains, but prevented the LPS-mediated reduction of cAMP response element binding protein (CREB) phosphorylation (Tomaz et al., 2020). CREB phosphorylation may exert a negative regulatory effect on NF-κB-mediated gene activation, thereby reducing cytokine gene expression (Snow and Albensi, 2016). Moclobemide, a reversible MAO-A selective inhibitor that is prescribed for the treatment of depression, was also tested for its anti-inflammatory effects using human whole blood. Blood samples were stimulated with LPS and phytohemagglutinin and moclobemide. Moclobemide reduced unstimulated TNF-α and IL-8 expression but significantly increased interleukin-10 expression in LPS-treated samples (Lin et al., 2000). Interleukin 10 is a major anti-inflammatory cytokine that inhibits lymphocyte and monocyte/macrophage pro-inflammatory cytokine expression (Opal and DePalo, 2000).

Parkinson’s disease is a progressive neurodegenerative disease characterized by resting tremor, muscular rigidity, and gait disturbances. Postmortem analysis of Parkinson’s patients showed significant microglial cell activation in the affected brain region and increased pro-inflammatory cytokine expression (Sawada et al., 2006; Subhramanyam et al., 2019). Microglial make up 10–15% of the glial cell population in adult brains and function as resident immune cells of the brain (Subhramanyam et al., 2019). Their function is multifaceted and exerts a neuroprotective and neurotoxic effect on neuronal cells. Upon activation, they express a number of pro-inflammatory cytokines such as IL-6, IL-1β, and TNF-α as well as nitric oxide and reactive oxygen molecules. In a rotenone-induced rat model of Parkinson’s disease, rasagiline reduced TNF-α mRNA expression in brain homogenates (Fernandez et al., 2011). In addition, daily administration of ladostigil (irreversible MAO-A/B inhibitor) to 16-month-old rats prevented the development of spatial memory deficits at 22 months of age and was associated with a significant decrease in gene expression of TNF-α, IL-6, and IL-1β in the parietal cortex (Panarsky et al., 2012). In complementary glial cell culture studies, ladostigil and its metabolites reduced LPS-induced TNF-α, IL-1β mRNA, and protein, and reduced LPS-induced degradation of IκB-α and nuclear translocation of NF-κB p65. In addition, ladostigil inhibited LPS-induced phosphorylation of p38 and ERK 1/2 mitogen-activated protein kinase (MAPK) (Panarsky et al., 2012). Parkinson’s disease patients have an increased risk of having a DJ-1 gene mutation (28511254). DJ-1 is an oxidative stress sensor that localizes to mitochondria. Downregulation of DJ-1 expression using shRNA increased cell sensitivity to dopamine as measured by elevated IL-1β and IL-6 expression. DJ-1 deficient microglia showed increased MAO activity and elevated intracellular reactive oxygen species (ROS) and nitric oxide. Treatment of cultures with rasagiline significantly reduced IL-1β, TNF-α, ROS, and nitric oxide levels (Trudler et al., 2014). Moclobemide also reduced LPS-induced IL-1β and TNF-α gene and protein expression in rat primary glial cell cultures and this was associated with reduced NF-κB p65 translocation to the nucleus (Bielecka et al., 2010). At odds with these findings, one study showed that phenelzine induced TNF-α and IL-6 expression in an NF-κB-dependent manner in LPS-activated microglial cells (Chung et al., 2012). This is unlikely to be reflective of this specific MAO-A/B inhibitor because phenelzine did reduce TNF-α expression in an *in vivo* animal model of LPS-induced periodontal disease (Ekuni et al., 2009).

Ischemia/reperfusion (I/R) tissue injury is a complex biological phenomenon that affects various tissues and organs and is dependent on the degree and length of time that blood flow is reduced. Cellular acidosis occurs, adenosine triphosphate generation is reduced, intracellular and mitochondrial calcium levels increase, and the regulation of cell volume is disrupted. Tissue reperfusion reinstates oxygen, yet the return of oxygen drives ROS generation. This increase in ROS generation induces cell and tissue damage and induces inflammation. Mitochondrial MAO is one of the enzymes that contribute to the overall generation of ROS molecules in the cell (Kalogeris et al., 2016). The brain, kidney, and heart are susceptible to ischemia/reperfusion tissue injury and MAO inhibitors reduced tissue injury in all of these models.

Nialamide (an irreversible MAO-A/B inhibitor) reduced neuroinflammation in a transient middle cerebral artery occlusion murine model. Specifically, nialamide reduced microglia and astrocytes numbers and TNF-α protein expression in the brain. Administration of nialamide even at 3 h post-ischemia effectively reduced neuronal injury and aided functional recovery (Liu et al., 2020). In a renal ischemia/reperfusion study, pargyline (irreversible MAO-B inhibitor) reduced IL-1β and TNF-α gene expression (Chaaya et al., 2011). The mechanism by which pargyline reduced cytokine expression was not examined. Nevertheless, it was previously established that renal ischemia/reperfusion is associated with a rapid increase in MAO-dependent hydrogen peroxide generation that occurs within 15 min of reperfusion. When pargyline was administered 15 min prior to ischemia, the hydrogen peroxide production was reduced (Kunduzova et al., 2002a; Kunduzova et al., 2002b). Ischemia/reperfusion injury in the heart is also associated with an increased expression of pro-inflammatory cytokines and chemokines (Frangogiannis, 2004; Najafi et al., 2018). Serotonin and catecholamines such as dopamine and norepinephrine play a significant role in cardiac function (Kaludercic et al., 2011; Kaludercic et al., 2014). Upregulation in monoamine oxidase activity due to increased substrate availability would result in an increase in hydrogen peroxide and aldehyde end products. These affect the transfer of electrons across the respiratory chain, which opens permeability transition pores that lead to cardiomyocyte death, oxidative damage, and heart failure. These finding support that monoamine oxidases may be a valid target for the management of I/R-associated cardiovascular damage (Deshwal et al., 2017). Changes in pro-inflammatory cytokine and chemokine expression by MAO inhibitors have not been directly examined in cardiac ischemia/reperfusion. Regardless, I/R-induced infarct size and infiltration of acute inflammatory cells in rat cardiac tissues were assayed for myeloperoxidase activity (i.e., neutrophil and monocytes recruitment), a marker of inflammation. Pargyline and clorgyline (irreversible MAO-A inhibitor) both reduced myeloperoxidase activity in cardiac tissues (Bianchi et al., 2005). MAO inhibitor-mediated reduction in cell recruitment may possibly be reflective of reduced chemokine expression.

Non-immune cells such as epithelial cells of mucosal tissues also express cytokines and chemokines in response to inflammation. Periodontal disease is a chronic inflammatory destructive oral mucosal disease that primarily occurs in response to Gram-negative bacteria in biofilm and the ensuing host immune response. In an LPS-induced rat model of periodontal disease, hydrogen peroxide and TNF-α significantly increased in the epithelium of diseased tissues. Adjunctive use of phenelzine reduced hydrogen peroxide, TNF-α, and reduced disease progression markers: epithelial cell proliferation, migration, and bone loss. In epithelial cell culture, deprenyl (aka selegiline; irreversible MAO-B inhibitor) and phenelzine reduced LPS-induced TNF-α protein expression. Smoking is also a potent driver of inflammation in lung mucosal tissues. Bronchial epithelial cells that are activated by cigarette smoke produce a wide variety of pro-inflammatory cytokines and chemokines (Barnes, 2016). In airway epithelial cell cultures, cigarette smoke medium induced oxidative stress and IL-8 protein expression and deprenyl reduced its expression (Cui et al., 2017). Cigarette smoke-containing medium increased oxidative stress (as measure by dihydrochlorofluorescein acetate) and this was reduced by deprenyl. Functionally, deprenyl fully reversed the effect of cigarette smoke on IκB kinase phosphorylation, degradation of IκB, and nuclear translocation of the NF-κB p65 subunit into the nucleus. In a cigarette smoke-induced chronic obstructive pulmonary disease model using rats, deprenyl (selegiline) reduced cigarette smoke-induced MAO-B activity. It did not alter total inflammatory cell infiltrate in bronchoalveolar lavage (BAL) nor did it reduce smoking-induced macrophage infiltrates in BAL. Even so, deprenyl significantly reduced BAL levels of cytokine-induced neutrophil chemoattractant, monocyte chemoattractant protein 1 and IL-6 in smoke-treated animals, and increased expression of the anti-inflammatory cytokine IL-10. These changes were associated with reduced phosphorylation of ERK1/2 and p38 MAPKs as well as reduced nuclear translocation of NF-kB p65 subunit in lung tissue extracts (Cui et al., 2020). MAO inhibitor reduction of cytokine expression may in part be explained by a reduction in oxidative stress molecules such as hydrogen peroxide. Indeed, cigarette smoke also has a number of aldehydes and these aldehydes induce pro-inflammatory cytokine expression (Sapkota and Wyatt, 2015). It is unclear whether the reduction of aldehydes due to MAO inhibitors may also play a role in the reduction of cytokine expression. In contrast to lung tissue, smoking reduced brain MAO-A levels relative to non-smokers. This inhibition of MAO-A in the brain by smoking may contribute to the difficulty of achieving cessation of smoking in patients with depression. Studies are needed to ascertain whether the level of MAO-A inhibition due to chronic smoking is associated with antidepressant effects (Fowler et al., 1996).

Altered MAO-A expression has been associated with high-grade prostate cancer (Gleason grade 4 and 5), tumorigenesis, metastasis, and poorer prognosis (Peehl et al., 2008; Wu et al., 2014; Liao et al., 2018; Shih, 2018; Yin et al., 2018). MAO-A protein and gene expression is increased in bone metastasis and inhibition of MAO-A activity with clorgyline reduced the onset of bone metastasis, metastasis burden, and mortality. Significantly, this reduction was associated with downregulation of sonic hedgehog signaling and stromal cell expression of IL-6 (Wu et al., 2017).

Inflammasomes are protein complexes that recognize inflammation-inducing signals and ultimately control the production of pro-inflammatory cytokines such as IL-1β and IL-18 (Strowig et al., 2012). Formation of inflammasome protein complex was triggered by MAO-B-induced ROS in LPS/ATP-stimulated murine bone marrow-derived macrophages and human monocyte-derived macrophages. MAO-B activity was required for the generation of ROS through a NF-κB-mediated mechanism. Inflammasome activation was associated with increased IL-1β gene and protein expression, and this was notably reduced with rasagiline co-treatment (Sanchez-Rodriguez et al., 2020).

Collectively, irreversible and reversible MAO-A, MAO-B, and MAO-A/B inhibitors have all been shown to significantly reduce inflammation-associated cytokine and chemokine gene and protein expression in a variety of cell culture and animal studies (Table 2). Understanding the regulatory mechanism(s) mediating these findings is critical to ascertain if MAO inhibitors are to be developed as anti-inflammatories.

## MAO Inhibitor–Mediated Reduction of Metabolic End Products

MAO-mediated degradation of amines generates hydrogen peroxide (H_2_O_2_), aldehyde, and ammonium as metabolic end products (Figure 1-middle right), and all of these end products may potentially impact inflammation. In a rat aorta vascular ring model, LPS induced MAO-A and MAO-B expression and treatment with MAO-A and MAO-B selective inhibitors significantly reduced H_2_O_2_ generation and this was associated with improved vascular function (Ratiu et al., 2018). Hydrogen peroxide plays a role in physiologic signaling that is needed to induce inflammation, but high concentrations of H_2_O_2_ may induce DNA damage and modify proteins, lipids, and other molecules (Wittmann et al., 2012; Winterbourn, 2013; Sturza et al., 2019). Hydrogen peroxide is an oxidative stress molecule associated with the generation of ROS-associated molecules. By the Fenton reaction, unquenched H_2_O_2_ can react with metal ions to form hydroxyl radicals (^●^OH) and hydroxide ions (OH^−^). These radicals are associated with irreversible oxidative damage in cellular targets (Oyewole and Birch-Machin, 2015; Pavlin et al., 2016). Hydroxyl radicals are highly reactive, their toxicity is non-selective and there are no enzymes to specifically detoxify it (Pavlin et al., 2016). Hydrogen peroxide can induce inflammation by activation of NFκB, a key regulatory molecule in inflammation (Wittmann et al., 2012). In gastric epithelial cells, hydrogen peroxide-induced expression of IL-8 gene and protein expression was mediated through NFκB activation (Kim et al., 2011). Overall, hydrogen peroxide generation is a key and needed regulator of inflammation, but increases can lead to significant mitochondrial damage and unwanted inflammation (van der Vliet and Janssen-Heininger, 2014; Deshwal et al., 2017). This overloading is thought to play an important role in the pathogenesis of a number of chronic and autoimmune inflammatory diseases (Di Dalmazi et al., 2016; Pravda, 2020).

**FIGURE 2:**
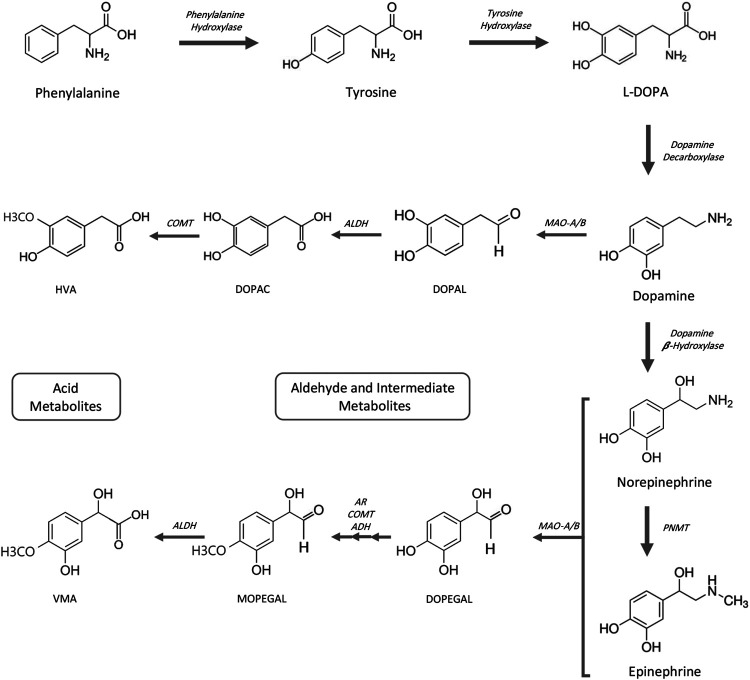
Catecholamine pathway for biosynthesis and MAO-mediated degradation. ADH, alcohol dehydrogenase; AR, aldehyde reductase; ALDH, aldehyde dehydrogenase; COMT, catechol-O-methyltransferase; DOPAC, 3,4-dihydroxyphenylacetic acid; DOPAL, 3,4-dihydroxyphenylacetaldehyde; DOPEGAL, 3,4-dihydroxyphenylglycolaldehyde; HVA, homovanillic acid; L-DOPA, L-3,4-dihydroxyphenylalanine; MAO-A/B, monoamine oxidases A and B; MOPEGAL, 3-methoxy-4-hydroxyphenylglycolaldehyde; PNMT, phenolethanolamine-N-methyltransferase; VMA, vanillylmandelic acid.

Generation of biogenic aldehydes has a significant effect on neuroinflammation associated with Alzheimer’s and cardiovascular disease-associated fibrosis, vascular changes, and cardiac hypertrophy (Pan et al., 2016; Nelson et al., 2017; Joshi et al., 2019). Specifically, MAO-dependent aldehyde generation induced mitochondrial dysfunction and ultimately led to heart failure in a pressure overload animal model (Deshwal et al., 2017). Biogenic aldehydes are formed by enzyme-dependent oxidation of primarily glucose, unsaturated lipids, and primary amines. Oxidative deamination of norepinephrine and dopamine by monoamine oxidase enzymes forms catecholaldehydes 3,4-dihydroxyphenylglycolaldehyde (DOPEGAL) and 3,4-dihydroxyphenylacetaldehyde (DOPAL), respectively, and hydrogen peroxide (Nelson et al., 2017). DOPAL—an aldehyde—is toxic because of its two reactive functional groups (catechol and aldehyde) that result in DOPAL-mediated protein cross-linking (Rees et al., 2009). DOPAL is further oxidized to 3,4-dihydrophenylacetic acid (DOPAC) *via* aldehyde dehydrogenase 2 (ALDH2) (Jinsmaa et al., 2009) (Figure 2). In an atherosclerotic plaque mouse model, ALDH2 gene silencing (increased aldehydes) was associated with increased phosphorylation of NF-kB p65, AP-1, and MAPK, and downstream expression of inflammatory molecules ICAM-1, MMP-2, IL-6, and MCP-1. Conversely, ALDH2-induced overexpression (decreased aldehydes) triggered the opposite effects (Pan et al., 2016). Altogether, increased aldehyde generation and accumulation can serve as an inflammatory driver.

Ammonia is also a by-product of MAO enzyme activity, although its direct effect on inflammation is less clear. In hepatic encephalopathy (HE), infection and inflammation play a role in its precipitation and development and it acts in concert with hyperammonemia. Liver injury is associated with concurrent hyperammonemia and immune system dysregulation and severity of HE was dependent on both aspects (Tranah et al., 2013). The significance of MAO enzyme induction of ammonium and its role in mediating disease-associated inflammation has not been examined. All things considered, MAO enzyme activity is associated with the generation of several metabolic end products. If these metabolic end products are increased beyond the biological capacity of the tissues, they may play a role in driving inflammation and ultimately disease. Reduction of these metabolites due to MAO inhibitors does at least in part explain some of their mechanism of action. However, MAO inhibitors—by their inhibition of MAO enzyme activity—significantly increase catecholamines and this increase also regulates inflammation.

## MAO Inhibitor–Mediated Increase in Catecholamine Substrates

Catecholamines are monoamines that include dopamine, norepinephrine (noradrenalin), and epinephrine (adrenalin), and all are derived from the amino acid tyrosine or indirectly from phenylalanine. Phenylalanine can be converted to tyrosine by phenylalanine hydroxylase (Ise et al., 1988). Tyrosine hydroxylation by tyrosine hydroxylase generates L-DOPA, which in turn is converted to dopamine by DOPA decarboxylase (also known as aromatic L-amino acid decarboxylase). Norepinephrine is synthesized from dopamine by hydroxylation with dopamine β-hydroxylase and epinephrine is subsequently synthesized from norepinephrine by phenylethanolamine-N-methyltransferase. Degradation of catecholamines begins primarily by monoamine oxidase-mediated deamination (Figure 2). Meanwhile, to a lesser degree, catechol-O-methyltransferase (COMT) methylation of catecholamines also occurs (not shown). Aldehyde reductase, alcohol dehydrogenase, and aldehyde dehydrogenase complete metabolic degradation to form homovanilic acid from dopamine and vanillyl mandelic acid from (nor)epinephrine (Eisenhofer et al., 2004; Fernstrom and Fernstrom, 2007; Jung-Klawitter and Kuseyri Hubschmann, 2019; Nolan and Gaskill, 2019; Goldstein, 2020).

These catecholamines are described as neurotransmitters and as hormones that regulate physiological processes and are associated with neurological, psychiatric, endocrine, and cardiovascular diseases (Eisenhofer et al., 2004). Furthermore, a third peripheral catecholaminergic system in which dopamine acts as an autocrine/paracrine mediator of local tissue function has also been described (Goldstein et al., 1995). In this model, dopamine within cells can be synthesized in several ways: from circulating DOPA that escapes from sympathetic nerves and adrenal medulla, tyrosine to DOPA conversion, or DOPA production by demethylation of circulating methoxytyrosine (Goldstein et al., 1995). DOPA synthesis from tyrosine in cells that express tyrosine hydroxylase has been described in a variety of immune cells (Jiang et al., 2006; Matt and Gaskill, 2020). Monocytes/macrophages synthesize catecholamines and—when stimulated with LPS—showed increased tyrosine hydroxylase expression and increased levels of dopamine, norepinephrine, and epinephrine (Cosentino et al., 2000; Brown et al., 2003; Flierl et al., 2007; Barnes et al., 2015). T cells, B cells, and polymorphonuclear leukocytes (PMNs) also express tyrosine hydroxylase and synthesize catecholamines (Harmony et al., 1975; Bergquist et al., 1994; Cosentino et al., 2000; Flierl et al., 2007). Rank order of intracellular dopamine and (nor)epinephrine levels showed that T- and B-lymphocytes were relatively equivalent and higher than monocytes, which were higher than granulocytes (Cosentino et al., 2000). Dopamine synthesis in non-immune cells has also been described. Dopamine synthesis by alveolar epithelial cells was reflective of L-DOPA cell uptake and intracellular conversion to dopamine by cellular aromatic L-amino acid decarboxylase (AADC) (Adir et al., 2004). Catecholamines do regulate immune responses. Stimulation of PMNs with (nor)epinephrine reduced markers of cell activation (Scanzano et al., 2015). Dopamine is recognized as a catecholamine that modulates immune responses (Pinoli et al., 2017; Matt and Gaskill, 2020). Dopamine reduced expression of interferon-γ by Con A-stimulated lymphocytes (Bergquist et al., 1994). Of interest, pargyline treatment of Concanavalin A-stimulated lymphocyte cultures significantly increased intracellular and supernatant levels of dopamine, norepinephrine, and epinephrine. Increased catecholamines were associated with attenuation of lymphocyte proliferation, and this process was regulated by catecholamine selective receptors (Qiu et al., 2005). Treatment of rat pheochromocytoma PC12 cells with an irreversible MAO-A inhibitor (clorgyline) and MAO-B inhibitors (rasagiline and deprenyl) increased dopamine and norepinephrine in the medium and cells (Goldstein et al., 2016). Local increase in pericellular catecholamines may regulate inflammation through adrenergic and dopaminergic receptor signaling pathways.

## Catecholamine Receptor–Mediated Signaling: Adrenergic Receptors

(Nor)epinephrine signaling is mediated by seven transmembrane GTP-protein coupled adrenergic receptors (ARs). These receptors activate GTP-binding regulatory G proteins and in turn are effectors of adenylyl cyclase and phospholipase C. ARs are classified into three major types (α_1_, α_2_, and β) and each is further divided into three subtypes (Strosberg, 1993; Scanzano and Cosentino, 2015). Granulocytes, monocytes/macrophages, mast cells, and lymphocytes express both α_1_-AR and β_2_-AR. Generally, stimulation of α_1_-AR and β_2_-AR expressed on immune cells can either drive or inhibit inflammation, respectively (Bellinger and Lorton, 2014; Scanzano and Cosentino, 2015). Blocking of α_2_-AR signaling was also associated with reduced expression of TNF-α in macrophages (Spengler et al., 1994; Barnes et al., 2015). Conversely, stimulation of β_2_-AR with agonists reduced TNF-α gene and protein expression in macrophages (Spengler et al., 1994) and activation of PMNs (Scanzano et al., 2015). Inhibition of LPS-induced TNF-α expression in rats by an anti-glaucoma drug (GLC756) was mediated by its antagonistic effect on α2 adrenoreceptors and its agonist effect on β2 adrenoreceptors (Laengle et al., 2006). Pargyline inhibition of lymphocyte proliferation was mediated by a drug-induced increase in intracellular and extracellular catecholamines and subsequent β-adrenoreceptor signaling and cAMP activation (Qiu et al., 2005). Ligation of β_2_-AR also interferes with activation of NF-κB, a major regulator of immunity and inflammation. The β_2_-AR inhibition of NF-κB phosphorylation, nuclear translocation, DNA binding, and transcriptional activity has been described in a variety of immune and non-immune cells, although the point of crosstalk between β_2_-AR and NF-κB varies; it can be positive or negative and is very cell-type specific and context-dependent (Kolmus et al., 2015). These data support that MAO inhibitor-mediated increase in catecholamines may mediate immune cell response and impact cytokine expression *via* adrenergic receptor-mediated signaling.

## Catecholamine Receptor–Mediated Signaling: Dopamine Receptors

Dopamine in the paracellular compartment can signal through a family of dopamine receptors (DRs) that belong to a large family of G protein-coupled receptors. There are five DRs that are grouped into two subclasses: D1-like class (DR1 and DR5) and D2-like class (DR2, DR3, and DR4) (Beaulieu and Gainetdinov, 2011; Beaulieu et al., 2015; Gurevich et al., 2016). In the CNS, DR1, DR2, and DR5 are most highly expressed but their distribution varies. In contrast, DR3 and DR4 are expressed at lower levels and are more limited. In non-CNS tissues, all receptor subtypes are detected but vary in relative expression based on the tissue examined (Beaulieu and Gainetdinov, 2011; Handa et al., 2019). Dopamine receptors can signal through multiple pathways. DR1-like receptors can couple with Gα_s/olf_ proteins and D2-like receptors are coupled to Gα_i/o_ proteins and they either stimulate or inhibit cAMP activation, respectively (Jage, 1989; Klein et al., 2019). Downstream D1-like class receptors in turn regulate protein kinase A (PKA) and exchange proteins activated by cAMP (EPAC 1 and EPAC2). Alternatively, DR1-like receptors can couple to G_αq_ to activate phospholipase C (PLC), leading to the formation of inositol trisphosphate (IP_3_) and diacylglycerol (DAG) and subsequent mobilization of Ca^+2^ and protein kinase C (PKC) activation (Beaulieu and Gainetdinov, 2011; Beaulieu et al., 2015). D2-like receptors inhibit cAMP activation but can signal through the G_βγ_ subunit, which in turn activates PLC and increases intracellular Ca^+2^. In addition, G protein-independent DR2 receptors regulate β-Arrestin 2 (βArr2) signaling. βArr2 acts as a molecular scaffold and plays a role in dopamine regulation of serine/threonine kinases Akt (protein kinase B) and glycogen synthase kinase 3 (GSK3) (Beaulieu and Gainetdinov, 2011; Beaulieu et al., 2015). Accordingly, dopamine binding to this family of receptors can signal through multiple pathways that in turn can mediate a variety of cellular responses. Cellular responses to dopamine are based on receptor type, relative expressions levels, and can change based on the state of cell activation (Pinoli et al., 2017; Matt and Gaskill, 2020). Therefore, any changes in the local concentration of dopamine may ultimately impact inflammatory responses.

## MAO Inhibitors: Catecholamines Regulate Osteoclastogenesis

Regulation of inflammation by MAO inhibitor-mediated increase of catecholamines has been examined from the perspective of osteoclastogenesis and bone loss. Osteoclasts—which ultimately regulate bone resorption—are derived from monocyte/macrophage hematopoietic cells (Teitelbaum and Ross, 2003; Koide et al., 2017; Furuya et al., 2018). Differentiation along the osteoclast lineage is driven by macrophage colony-stimulating factor (M-CSF) and the receptor activator of nuclear factor-kappa β ligand (RANKL). RANKL is expressed by osteoblasts, T- and B-lymphocytes, stromal and osteocyte cells, and fibroblasts (Teitelbaum and Ross, 2003; Di Benedetto et al., 2013; Xiong et al., 2015). RANKL binding to its receptor on the pre-osteoclast cell surface triggers activation of nuclear factor of activated T cells, cytoplasmic tail 1 (NFATc1), and c-Fos transcription factors, which are required for osteoclastogenesis (Teitelbaum and Ross, 2003). RANKL-mediated differentiation is inhibited by the RANKL decoy receptor, osteoprotegerin (OPG) (Teitelbaum and Ross, 2003; Gruber, 2019). A balance between RANKL and OPG regulates osteoclast-mediated bone loss; however, inflammatory bone diseases are associated with increased osteoclast differentiation and function and ultimately bone loss.

Phenelzine reduced rat periodontal disease-associated bone loss but the mechanism regulating this effect was not explored (Ekuni et al., 2009). Tranylcypromine reduced M-CSF and RANKL-mediated bone marrow monocyte (BMM) osteoclast differentiation and function. This reduction was *via* reduced Akt, NFATc1, and c-fos signaling (Liu et al., 2019). Although MAO-A and MAO-B were detected in BMM cells, only MAO-A expression was either induced or reduced by RANKL and tranylcypromine, respectively. Tranylcypromine reduced MAO-A activity and MAO-A knockdown reduced osteoclast number and function (Liu et al., 2019). Tranylcypromine reduced LPS-mediated calvarial bone loss and osteoclast numbers in mice. In addition, tranylcypromine reversed both osteoblast decrease and osteoclast increase in ovariectomized mice and improved femur bending stiffness and strength, elastic modulus, and maximum bending strength (Liu et al., 2019).

Understanding the mechanism by which MAO inhibitors mediate their effect on osteoclast differentiation and function has not been fully elucidated. Accumulation of mitochondrial hydrogen peroxide is a potent driver of osteoclast differentiation and bone loss in ovariectomized mice and its reduction reduced bone loss (Bartell et al., 2014). Nonetheless, it was recently shown that MAO metabolism of dopamine did not increase cytosolic hydrogen peroxide levels, but in fact leads to increased electron chain activity (Graves et al., 2020). These data suggest that catecholamine increase may possibly regulate osteoclast signaling. Dopamine/DR signaling does have an important regulatory role in osteoclasts differentiation and function but this is dopamine-receptor specific (Nakashioya et al., 2011; Hanami et al., 2013a; Hanami et al., 2013b; Yang et al., 2016; Motyl et al., 2017; Handa et al., 2019; Wang et al., 2021). No significant effect on osteoclast differentiation and function was found when human and mouse monocyte cultures were treated with a DR1-agonist (Hanami et al., 2013a; Hanami et al., 2013b; Yang et al., 2016; Wang et al., 2021; Table 3). Treatment of cultures with a DR1-antagonist had primarily no effect on osteoclastogenesis (Hanami et al., 2013a; Yang et al., 2016; Wang et al., 2021), although one study did show DR1-antagonist inhibition of osteoclastogenesis (Nakashioya et al., 2011). In contrast, human and mouse osteoclast differentiation and function were significantly inhibited with DR2-like agonists. In addition, dopamine-mediated inhibition of osteoclastogenesis was negated by DR2-like receptor antagonists (Hanami et al., 2013a; Hanami et al., 2013b; Yang et al., 2016; Handa et al., 2019; Wang et al., 2021). Dopamine regulation of osteoclastogenesis through DR2 signaling inhibited cAMP activation and reduced activation of PKA and CREB signaling (Handa et al., 2019). Taken together, dopamine regulates osteoclast differentiation and DR2 appears to play a critical role. DRs vary in their affinity for dopamine and signaling can be changed based on relative dopamine concentration (Melnikov et al., 2020). Whether an MAO inhibitor-mediated increase in dopamine drives altered DR signaling in the context of osteoclastogenesis requires further investigation.

**TABLE 3:** Dopamine receptor regulation of osteoclast differentiation and function.

Targeted dopamine receptor	Agents	Activation or inhibition	Cellular responses	Cell populations	References
D1-like receptor	SCH23390	Antagonist	Inhibition of osteoclast differentiation and suppression the pathology of CIA and RA in animal model	Mouse bone marrow–derived macrophage	Nakashioya et al. (2011)
D2-like receptor	–	Agonist	Inhibition of osteoclast differentiation and function	Human monocytes	Hanami et al. (2013b)
D1-like receptor	–	Agonist	No effect on osteoclast differentiation		
D2-like receptor	Pramipexole	Agonist	Inhibition of LPS-induced osteoclast differentiation and function	Human PBMC monocytes	Hanami et al. (2013a)
D1-like receptor	SKF38393	Agonist	No effect on osteoclast differentiation		
	SCH23390	Antagonist	No effect on osteoclast differentiation		
D2-like receptor	Quinpirole	Agonist	Inhibition of osteoclast differentiation and function	Mouse bone marrow–derived monocytes	Yang et al. (2016)
	Haloperidol	Antagonist	Reduction of dopamine inhibition of osteoclast function		
D1-like receptor	SKF38393	Agonist	No effect on osteoclast differentiation		
	SCH23390	Antagonist			
D2-like receptor	Quinpirole	Agonist	Inhibition of osteoclastogenesis mediated signaling	Mouse monocytes (RAW 264.7)	Wang et al. (2021)
	Haloperidol	Antagonist	Negates the inhibitory effect of dopamine on osteoclastogenesis		
D1-like receptor	SKF38393	Agonist	No effect on osteoclast differentiation		
	SCH23390	Antagonist			
D2-like receptor	Pramipexole ropinirole bromocriptine	Agonist	Inhibition of osteoclast function	Mouse bone marrow–derived monocytes	Handa et al. (2019)

## MAO Inhibitors: Joint Inflammatory Diseases

A historical clinical report that described nine patients who were treated with MAO inhibitors (tranylcypromine, phenelzine, and isocarboxazid) for their depression also reported a significant reduction of joint pain and stiffness. Four of these patients had rheumatoid arthritis (Lieb, 1983). Rheumatoid arthritis (RA) is a chronic autoimmune inflammatory joint disease causing cartilage and bone destruction. Joint swelling reflects synovial membrane inflammation subsequent to infiltration by leukocytes, innate, and adaptive immune cells (Smolen et al., 2016). The immune cell-rich environment is associated with expression of inflammatory cytokines and chemokines such as TNF-α and IL-6. Activated fibroblasts in association with activated T and B cells, monocytes, and macrophages ultimately drive osteoclast differentiation *via* expression of RANKL. Osteoclasts form bony erosions beginning at the junction of cartilage, periosteal synovial membrane insertion, and bone (Scott et al., 2010; Smolen et al., 2016).

Arthritic joint inflammation has been examined using [^11^C]-D-deprenyl, an enantiomer of L-deprenyl (selegiline). In comparison to L-deprenyl, D-deprenyl is a less potent inhibitor of MAO-B and inhibits MAO-A and has been used as a radioligand tracer with positron emission tomography (PET) imaging (Knoll and Magyar, 1972; Robinson, 1985). PET imaging of RA patients’ inflamed knee joints showed high tracer uptake in the inflamed joint synovium and this uptake was reduced 50% after glucocorticoid treatment (Danfors et al., 1997). This uptake was not solely specific to arthritic diseases because subsequent studies showed D-deprenyl tracer uptake in inflamed joints that were associated with traumatic ankle sprains and whiplash-associated disorders. In both cases, tracer uptake was localized to joint-associated soft tissues (Linnman et al., 2011; Aarnio et al., 2017). Patients experiencing persistent pain had prolonged D-deprenyl uptake (Aarnio et al., 2017). Frozen synovial membranes collected from RA and osteoarthritis (OA) patients showed MAO-B protein expression; this did not change with the inflammatory grade but relative binding of D-deprenyl did correlate with synovial inflammation (Lesniak et al., 2018). High-throughput enzyme screening assay identified that MAO-B was the most likely primary binding target of D-deprenyl but binding to MAO-A and angiotensin-converting enzyme (ACE) was also identified (Lesniak et al., 2016). Clarifying MAO isotypes and defining which cell population(s) in inflamed joints express MAO protein need further study.

Cells isolated from synovial tissues from RA and OA patients were found to be tyrosine hydroxylase (required for catecholamine synthesis) and VMAT2 positive (required for vesicular storage of catecholamines) (Capellino et al., 2010; Thomas Broome et al., 2020). Tyrosine hydroxylase (TH) positive cells were not found in control tissues. In RA and OA samples, TH positive cells were identified as macrophages, B cells, and fibroblasts. In RA samples, mast cells and neutrophils were also TH positive. Treatment of the cultures for short periods with reserpine inhibited TNF-α expression in these cells and local administration of reserpine improved the clinical score of collagen type II-induced arthritis in mice. Treatment of immune cells with reserpine reduced intracellular and increased extracellular catecholamines in cell culture medium at 24 and 48 h (Cosentino et al., 1999; Cosentino et al., 2000). However, a single short-term treatment with reserpine may increase cytoplasmic levels of catecholamines for a few hours (Lundborg, 1969; Capellino et al., 2010). These data support that localized peri (cellular) increase in catecholamines can dampen inflammation (Lundborg, 1969; Capellino et al., 2010). The effect of MAO inhibitors was examined in synovial cell cultures that were maintained under hypoxic conditions. Hypoxia induced tyrosine hydroxylase and catecholamine synthesis and reduced TNF-α expression. Blocking tyrosine hydroxylase (i.e., decreasing catecholamine synthesis) negated this effect and most significantly, the MAO-A/B inhibitor bifemelane (reversible MAO-A and irreversible MAO-B inhibitor) and a COMT inhibitor (collectively increasing catecholamine levels) significantly reduced TNF-α (Jenei-Lanzl et al., 2015). Early changes in intracellular catecholamine levels were not examined but at 24 h there was a significant increase in dopamine and norepinephrine levels in the cell culture supernatant (Jenei-Lanzl et al., 2015). Expression of pro-inflammatory cytokines such as TNF-α and IL-6 are critical drivers of synovial inflammation (Smolen et al., 2016).

Dopamine receptors do vary in their expression and relative affinity for dopamine so it is quite feasible that increased dopamine can trigger different DRs (Melnikov et al., 2020). DR2 agonists have shown a positive impact on joint inflammation but results vary based on the study and agonists examined (Capellino, 2020). A potential receptor-independent mechanism by which intracellular dopamine can mediate inflammation has also been described. In microglial cell cultures, dopamine attenuated LPS-induced expression of TNF-α, IL-1β, and IL-6 was not blocked by D1-like and D2-like receptor antagonists nor were they mimicked by D1-like and D2-like agonists (Yoshioka et al., 2020). However, dopamine attenuation of LPS-induced cytokine expression and NF-κB translocation to the nucleus occurred through formation of dopamine quinone (Yoshioka et al., 2020). Dopamine quinone forms by its auto-oxidation and covalent conjugation to cysteine residue sulfhydryl groups on proteins and results in the formation of quinoproteins (Sulzer and Zecca, 2000). Dopamine attenuation of LPS-induced cytokine expression was due to the formation of dopamine quinone (Yoshioka et al., 2020).

## MAO Inhibitor Drug Development

A review of monoamine oxidase inhibitor patents from 2012–2017 outlined a growing number of compounds that have been licensed as MAO inhibitors. These include novel synthetic compounds, natural compounds that have MAO inhibitory effects but with less restrictive side effects, and development of dual or multi-targeted inhibitors to target multifactorial diseases (Carradori et al., 2012; Carradori and Petzer, 2015; Carradori et al., 2018). Development of novel reversible and selective MAO-B inhibitors with reduced penetration into the CNS is one strategy that has been pursued to reduce CNS and diet-associated side effects (Gealageas et al., 2018). Repurposing of MAO inhibitor drugs is being tested for a variety of non-CNS diseases such as hair growth, ocular disease, muscular dystrophy (Menazza et al., 2010; Vitiello et al., 2018), sexual dysfunction, cardiovascular disease (Deshwal et al., 2017), and cancer (Shih, 2018). New and known coumarins have been synthesized and several have distinct antioxidant and anti-inflammatory effects. In particular, coumarin-stilbene analogs show selective MAO-B inhibitory effects (Detsi et al., 2017). All in all, MAO inhibitors are being actively developed for a wide spectrum of novel therapeutic uses.

## Concluding Remarks and Future Perspectives

MAO inhibitors have a long history of successful clinical use to manage CNS diseases such as depression, Parkinson’s, and Alzheimer’s. There is also an abundance of literature showing that MAO-A, MAO-B, and MAO-A/B inhibitors have CNS and non-CNS-associated anti-inflammatory effects in diseases of epithelial and soft and hard connective tissues. These data support their anti-inflammatory effects, but a concerted effort is needed to help focus which chronic inflammatory diseases are the best targets to select for expanded clinical testing. In conjunction, examining MAO-A and MAO-B expression in healthy and affected tissues and in recruited immune cells is needed to better understand the role(s) of MAOs in disease pathogenesis and will help to direct the selection of the most appropriate MAO inhibitors to test. Associated with these studies is a need to expand mechanistic studies as well. MAO inhibitors have broad-reaching cellular effects. They reduce metabolic end products such as hydrogen peroxide and aldehyde, and this reduction clearly has anti-inflammatory effects. Notwithstanding, MAO inhibitors also increase cellular and pericellular catecholamine levels. Catecholamines such as dopamine, norepinephrine, and epinephrine can be synthesized, stored, and released by immune and non-immune cells, and catecholamine signaling is associated with a reduction of inflammation. MAO inhibitors can increase both intracellular and extracellular catecholamines, and both can impact signaling. An increase in intracellular catecholamines may drive receptor-independent signaling. Alternatively, an increase in pericellular catecholamines may signal in a receptor-dependent manner. The diverse adrenergic and dopaminergic receptor families have different and divergent effects on inflammation and selective stimulation through their respective subtypes, and can dramatically alter the inflammatory response. When MAO inhibitors are being tested for therapeutic efficacy in inflammatory disease models, it is critical to expand mechanistic studies to examine these divergent signaling mechanisms. The prospect of either repurposing existing or developing novel MAO inhibitors for the management of chronic inflammatory diseases is a promising and exciting area of investigation.

## References

[B1] Aarnio M. Appel L. Fredrikson M. Gordh T. Wolf O. Sorensen J. (2017). Visualization of Painful Inflammation in Patients with Pain after Traumatic Ankle Sprain Using [(11)C]-D-deprenyl PET/CT. Scand. J. Pain 17, 418–424. 10.1016/j.sjpain.2017.10.008 29126847

[B2] Adir Y. Azzam Z. S. Lecuona E. Leal S. Pesce L. Dumasius V. (2004). Augmentation of Endogenous Dopamine Production Increases Lung Liquid Clearance. Am. J. Respir. Crit. Care Med. 169 (6), 757–763. 10.1164/rccm.200207-744OC 14701706

[B3] Al-Nuaimi S. K. Mackenzie E. M. Baker G. B. (2012). Monoamine Oxidase Inhibitors and Neuroprotection: a Review. Am. J. Ther. 19 (6), 436–448. 10.1097/MJT.0b013e31825b9eb5 22960850

[B4] Anderson M. C. Hasan F. McCrodden J. M. Tipton K. F. (1993). Monoamine Oxidase Inhibitors and the Cheese Effect. Neurochem. Res. 18 (11), 1145–1149. 10.1007/BF00978365 8255365

[B5] Balkwill F. (2009). Tumour Necrosis Factor and Cancer. Nat. Rev. Cancer 9 (5), 361–371. 10.1038/nrc2628 19343034

[B6] Balsa M. D. Gomez N. Unzeta M. (1989). Characterization of Monoamine Oxidase Activity Present in Human Granulocytes and Lymphocytes. Biochim. Biophys. Acta 992 (2), 140–144. 10.1016/0304-4165(89)90002-0 2503040

[B7] Barnes M. A. Carson M. J. Nair M. G. (2015). Non-traditional Cytokines: How Catecholamines and Adipokines Influence Macrophages in Immunity, Metabolism and the Central Nervous System. Cytokine. 72 (2), 210–219. 10.1016/j.cyto.2015.01.008 25703786 PMC4590987

[B8] Barnes P. J. (2016). Inflammatory Mechanisms in Patients with Chronic Obstructive Pulmonary Disease. J. Allergy Clin. Immunol. 138 (1), 16–27. 10.1016/j.jaci.2016.05.011 27373322

[B9] Bartell S. M. Kim H. N. Ambrogini E. Han L. Iyer S. Serra Ucer S. (2014). FoxO Proteins Restrain Osteoclastogenesis and Bone Resorption by Attenuating H2O2 Accumulation. Nat. Commun. 5, 3773. 10.1038/ncomms4773 24781012 PMC4015330

[B10] Beaulieu J. M. Espinoza S. Gainetdinov R. R. (2015). Dopamine Receptors - IUPHAR Review 13. Br. J. Pharmacol. 172 (1), 1–23. 10.1111/bph.12906 25671228 PMC4280963

[B11] Beaulieu J. M. Gainetdinov R. R. (2011). The Physiology, Signaling, and Pharmacology of Dopamine Receptors. Pharmacol. Rev. 63 (1), 182–217. 10.1124/pr.110.002642 21303898

[B12] Bellinger D. L. Lorton D. (2014). Autonomic Regulation of Cellular Immune Function. Auton. Neurosci. 182, 15–41. 10.1016/j.autneu.2014.01.006 24685093

[B13] Bergquist J. Tarkowski A. Ekman R. Ewing A. (1994). Discovery of Endogenous Catecholamines in Lymphocytes and Evidence for Catecholamine Regulation of Lymphocyte Function via an Autocrine Loop. Proc. Natl. Acad. Sci. U S A. 91 (26), 12912–12916. 10.1073/pnas.91.26.12912 7809145 PMC45550

[B14] Bianchi P. Kunduzova O. Masini E. Cambon C. Bani D. Raimondi L. (2005). Oxidative Stress by Monoamine Oxidase Mediates Receptor-independent Cardiomyocyte Apoptosis by Serotonin and Postischemic Myocardial Injury. Circulation. 112 (21), 3297–3305. 10.1161/CIRCULATIONAHA.104.528133 16286591

[B15] Bielecka A. M. Paul-Samojedny M. Obuchowicz E. (2010). Moclobemide Exerts Anti-inflammatory Effect in Lipopolysaccharide-Activated Primary Mixed Glial Cell Culture. Naunyn Schmiedebergs Arch. Pharmacol. 382 (5-6), 409–417. 10.1007/s00210-010-0535-4 20811738

[B16] Billett E. E. (2004). Monoamine Oxidase (MAO) in Human Peripheral Tissues. Neurotoxicology. 25 (1-2), 139–148. 10.1016/S0161-813X(03)00094-9 14697888

[B17] Binda C. Mattevi A. Edmondson D. E. (2011). Structural Properties of Human Monoamine Oxidases A and B. Int. Rev. Neurobiol. 100, 1–11. 10.1016/B978-0-12-386467-3.00001-7 21971000

[B18] Blackwell B. (1963). Hypertensive Crisis Due to Monoamine-Oxidase Inhibitors. Lancet. 2 (7313), 849–850. 10.1016/s0140-6736(63)92743-0 14056007

[B19] Borish L. C. Steinke J. W. (2003). 2. Cytokines and Chemokines. J. Allergy Clin. Immunol. 111 (2 Suppl. l), S460–S475. 10.1067/mai.2003.108 12592293

[B20] Bortolato M. Chen K. Shih J. C. (2008). Monoamine Oxidase Inactivation: from Pathophysiology to Therapeutics. Adv. Drug Deliv. Rev. 60 (13-14), 1527–1533. 10.1016/j.addr.2008.06.002 18652859 PMC2630537

[B21] Bortolato M. Floris G. Shih J. C. (2018). From Aggression to Autism: New Perspectives on the Behavioral Sequelae of Monoamine Oxidase Deficiency. J. Neural Transm. (Vienna). 125 (11), 1589–1599. 10.1007/s00702-018-1888-y 29748850 PMC6215718

[B22] Bortolato M. Godar S. C. Davarian S. Chen K. Shih J. C. (2009). Behavioral Disinhibition and Reduced Anxiety-like Behaviors in Monoamine Oxidase B-Deficient Mice. Neuropsychopharmacology. 34 (13), 2746–2757. 10.1038/npp.2009.118 19710633 PMC2783894

[B23] Bortolato M. Shih J. C. (2011). Behavioral Outcomes of Monoamine Oxidase Deficiency: Preclinical and Clinical Evidence. Int. Rev. Neurobiol. 100, 13–42. 10.1016/B978-0-12-386467-3.00002-9 21971001 PMC3371272

[B24] Brown S. W. Meyers R. T. Brennan K. M. Rumble J. M. Narasimhachari N. Perozzi E. F. (2003). Catecholamines in a Macrophage Cell Line. J. Neuroimmunol. 135 (1-2), 47–55. 10.1016/s0165-5728(02)00435-6 12576223

[B25] Brunner H. G. Nelen M. R. van Zandvoort P. Abeling N. G. van Gennip A. H. Wolters E. C. (1993). X-linked Borderline Mental Retardation with Prominent Behavioral Disturbance: Phenotype, Genetic Localization, and Evidence for Disturbed Monoamine Metabolism. Am. J. Hum. Genet. 52 (6), 1032–1039. 8503438 PMC1682278

[B26] Campbell L. M. Maxwell P. J. Waugh D. J. (2013). Rationale and Means to Target Pro-inflammatory Interleukin-8 (CXCL8) Signaling in *Cancer* . Pharmaceuticals (Basel). 6 (8), 929–959. 10.3390/ph6080929 24276377 PMC3817732

[B27] Cao Q. Harris D. C. Wang Y. (2015). Macrophages in Kidney Injury, Inflammation, and Fibrosis. Physiology (Bethesda). 30 (3), 183–194. 10.1152/physiol.00046.2014 25933819

[B28] Capellino S. Cosentino M. Wolff C. Schmidt M. Grifka J. Straub R. H. (2010). Catecholamine-producing Cells in the Synovial Tissue during Arthritis: Modulation of Sympathetic Neurotransmitters as New Therapeutic Target. Ann. Rheum. Dis. 69 (10), 1853–1860. 10.1136/ard.2009.119701 20498218

[B29] Capellino S. (2020). Dopaminergic Agents in Rheumatoid Arthritis. J. Neuroimmune Pharmacol. 15 (1), 48–56. 10.1007/s11481-019-09850-5 31016462 PMC7136314

[B30] Carradori S. Petzer J. P. (2015). Novel Monoamine Oxidase Inhibitors: a Patent Review (2012 - 2014). Expert Opin. Ther. Pat. 25 (1), 91–110. 10.1517/13543776.2014.982535 25399762

[B31] Carradori S. Secci D. Bolasco A. Chimenti P. D'Ascenzio M. (2012). Patent-related Survey on New Monoamine Oxidase Inhibitors and Their Therapeutic Potential. Expert Opin. Ther. Pat. 22 (7), 759–801. 10.1517/13543776.2012.698613 22702491

[B32] Carradori S. Secci D. Petzer J. P. (2018). MAO Inhibitors and Their Wider Applications: a Patent Review. Expert Opin. Ther. Pat. 28 (3), 211–226. 10.1080/13543776.2018.1427735 29324067

[B33] Cathcart M. K. Bhattacharjee A. (2014). Monoamine Oxidase A (MAO-A): a Signature Marker of Alternatively Activated Monocytes/macrophages. Inflamm. Cel Signal. 1 (4). 10.14800/ics.161 PMC4457461 26052543

[B34] Chaaya R. Alfarano C. Guilbeau-Frugier C. Coatrieux C. Kesteman A. S. Parini A. (2011). Pargyline Reduces Renal Damage Associated with Ischaemia-Reperfusion and Cyclosporin. Nephrol. Dial. Transpl. 26 (2), 489–498. 10.1093/ndt/gfq445 20667995

[B35] Chaitidis P. Billett E. E. O'Donnell V. B. Fajardo A. B. Fitzgerald J. Kuban R. J. (2004). Th2 Response of Human Peripheral Monocytes Involves Isoform-specific Induction of Monoamine Oxidase-A. J. Immunol. 173 (8), 4821–4827. 10.4049/jimmunol.173.8.4821 15470022

[B36] Chaitidis P. Billett E. Kuban R. J. Ungethuem U. Kuhn H. (2005). Expression Regulation of MAO Isoforms in Monocytic Cells in Response to Th2 Cytokines. Med. Sci. Monit. 11 (8), BR259–265. 16049371

[B37] Chen L. Deng H. Cui H. Fang J. Zuo Z. Deng J. (2018). Inflammatory Responses and Inflammation-Associated Diseases in Organs. Oncotarget. 9 (6), 7204–7218. 10.18632/oncotarget.23208 29467962 PMC5805548

[B38] Chung H. S. Kim H. Bae H. (2012). Phenelzine (Monoamine Oxidase Inhibitor) Increases Production of Nitric Oxide and Proinflammatory Cytokines via the NF-kappaB Pathway in Lipopolysaccharide-Activated Microglia Cells. Neurochem. Res. 37 (10), 2117–2124. 10.1007/s11064-012-0833-y 22763802

[B39] Chung H. Y. Cesari M. Anton S. Marzetti E. Giovannini S. Seo A. Y. (2009). Molecular Inflammation: Underpinnings of Aging and Age-Related Diseases. Ageing Res. Rev. 8 (1), 18–30. 10.1016/j.arr.2008.07.002 18692159 PMC3782993

[B40] Cosentino M. Bombelli R. Ferrari M. Marino F. Rasini E. Maestroni G. J. (2000). HPLC-ED Measurement of Endogenous Catecholamines in Human Immune Cells and Hematopoietic Cell Lines. Life Sci. 68 (3), 283–295. 10.1016/s0024-3205(00)00937-1 11191644

[B41] Cosentino M. Marino F. Bombelli R. Ferrari M. Lecchini S. Frigo G. (1999). Endogenous Catecholamine Synthesis, Metabolism, Storage and Uptake in Human Neutrophils. Life Sci. 64 (11), 975–981. 10.1016/s0024-3205(99)00023-5 10201646

[B42] Cui Y. Liu K. W. K. Ip M. S. M. Liang Y. Mak J. C. W. (2020). Protective Effect of Selegiline on Cigarette Smoke-Induced Oxidative Stress and Inflammation in Rat Lungs In Vivo. Ann. Transl Med. 8 (21), 1418. 10.21037/atm-20-2426 33313163 PMC7723576

[B43] Cui Y. Liu K. W. Liang Y. Ip M. S. Mak J. C. (2017). Inhibition of Monoamine Oxidase-B by Selegiline Reduces Cigarette Smoke-Induced Oxidative Stress and Inflammation in Airway Epithelial Cells. Toxicol. Lett. 268, 44–50. 10.1016/j.toxlet.2017.01.005 28108387

[B44] Danfors T. Bergstrom M. Feltelius N. Ahlstrom H. Westerberg G. Langstrom B. (1997). Positron Emission Tomography with 11C-D-Deprenyl in Patients with Rheumatoid Arthritis. Evaluation of Knee Joint Inflammation before and after Intra-articular Glucocorticoid Treatment. Scand. J. Rheumatol. 26 (1), 43–48. 10.3109/03009749709065663 9057801

[B45] Deshwal S. Di Sante M. Di Lisa F. Kaludercic N. (2017). Emerging Role of Monoamine Oxidase as a Therapeutic Target for Cardiovascular Disease. Curr. Opin. Pharmacol. 33, 64–69. 10.1016/j.coph.2017.04.003 28528298

[B46] Detsi A. Kontogiorgis C. Hadjipavlou-Litina D. (2017). Coumarin Derivatives: an Updated Patent Review (2015-2016). Expert Opin. Ther. Pat. 27 (11), 1201–1226. 10.1080/13543776.2017.1360284 28756713

[B47] Di Benedetto A. Gigante I. Colucci S. Grano M. (2013). Periodontal Disease: Linking the Primary Inflammation to Bone Loss. Clin. Dev. Immunol. 2013, 503754. 10.1155/2013/503754 23762091 PMC3676984

[B48] Di Dalmazi G. Hirshberg J. Lyle D. Freij J. B. Caturegli P. (2016). Reactive Oxygen Species in Organ-specific Autoimmunity. Auto Immun. Highlights 7 (1), 11. 10.1007/s13317-016-0083-0 27491295 PMC4974204

[B49] Dinarello C. A. (2018). Overview of the IL-1 Family in Innate Inflammation and Acquired Immunity. Immunol. Rev. 281 (1), 8–27. 10.1111/imr.12621 29247995 PMC5756628

[B50] Dlugos A. M. Palmer A. A. de Wit H. (2009). Negative Emotionality: Monoamine Oxidase B Gene Variants Modulate Personality Traits in Healthy Humans. J. Neural Transm. (Vienna). 116 (10), 1323–1334. 10.1007/s00702-009-0281-2 19657584 PMC3653168

[B51] Edmondson D. E. Binda C. (2018). Monoamine Oxidases. Subcell Biochem. 87, 117–139. 10.1007/978-981-10-7757-9_5 29464559

[B52] Eisenhofer G. Kopin I. J. Goldstein D. S. (2004). Catecholamine Metabolism: a Contemporary View with Implications for Physiology and Medicine. Pharmacol. Rev. 56 (3), 331–349. 10.1124/pr.56.3.1 15317907

[B53] Ekuni D. Firth J. D. Nayer T. Tomofuji T. Sanbe T. Irie K. (2009). Lipopolysaccharide-induced Epithelial Monoamine Oxidase Mediates Alveolar Bone Loss in a Rat Chronic Wound Model. Am. J. Pathol. 175 (4), 1398–1409. 10.2353/ajpath.2009.090108 19779138 PMC2751537

[B54] Fernandez M. Negro S. Slowing K. Fernandez-Carballido A. Barcia E. (2011). An Effective Novel Delivery Strategy of Rasagiline for Parkinson's Disease. Int. J. Pharm. 419 (1-2), 271–280. 10.1016/j.ijpharm.2011.07.029 21807080

[B55] Fernstrom J. D. Fernstrom M. H. (2007). Tyrosine, Phenylalanine, and Catecholamine Synthesis and Function in the Brain. J. Nutr. 137 (6 Suppl. 1), 1539S–1547S; discussion 1548S. 10.1093/jn/137.6.1539S 17513421

[B56] Flierl M. A. Rittirsch D. Nadeau B. A. Chen A. J. Sarma J. V. Zetoune F. S. (2007). Phagocyte-derived Catecholamines Enhance Acute Inflammatory Injury. Nature. 449 (7163), 721–725. 10.1038/nature06185 17914358

[B57] Foong A. L. Grindrod K. A. Patel T. Kellar J. (2018). Demystifying Serotonin Syndrome (Or Serotonin Toxicity). Can. Fam. Physician 64 (10), 720–727. 30315014 PMC6184959

[B58] Fowler J. S. Volkow N. D. Wang G. J. Pappas N. Logan J. Shea C. (1996). Brain Monoamine Oxidase A Inhibition in Cigarette Smokers. Proc. Natl. Acad. Sci. U S A. 93 (24), 14065–14069. 10.1073/pnas.93.24.14065 8943061 PMC19495

[B59] Frangogiannis N. G. (2004). The Role of the Chemokines in Myocardial Ischemia and Reperfusion. Curr. Vasc. Pharmacol. 2 (2), 163–174. 10.2174/1570161043476375 15320517

[B60] Furuya M. Kikuta J. Fujimori S. Seno S. Maeda H. Shirazaki M. (2018). Direct Cell-Cell Contact between Mature Osteoblasts and Osteoclasts Dynamically Controls Their Functions In Vivo. Nat. Commun. 9 (1), 300. 10.1038/s41467-017-02541-w 29352112 PMC5775424

[B61] Gabay C. Kushner I. (1999). Acute-phase Proteins and Other Systemic Responses to Inflammation. N. Engl. J. Med. 340 (6), 448–454. 10.1056/NEJM199902113400607 9971870

[B62] Gaweska H. Fitzpatrick P. F. (2011). Structures and Mechanism of the Monoamine Oxidase Family. Biomol. Concepts. 2 (5), 365–377. 10.1515/BMC.2011.030 22022344 PMC3197729

[B63] Gealageas R. Devineau A. So P. P. L. Kim C. M. J. Surendradoss J. Buchwalder C. (2018). Development of Novel Monoamine Oxidase-B (MAO-B) Inhibitors with Reduced Blood-Brain Barrier Permeability for the Potential Management of Noncentral Nervous System (CNS) Diseases. J. Med. Chem. 61 (16), 7043–7064. 10.1021/acs.jmedchem.7b01588 30016860

[B64] Gillman P. K. (2018). A Reassessment of the Safety Profile of Monoamine Oxidase Inhibitors: Elucidating Tired Old Tyramine Myths. J. Neural Transm. (Vienna). 125 (11), 1707–1717. 10.1007/s00702-018-1932-y 30255284

[B65] Goldstein D. S. Jinsmaa Y. Sullivan P. Holmes C. Kopin I. J. Sharabi Y. (2016). Comparison of Monoamine Oxidase Inhibitors in Decreasing Production of the Autotoxic Dopamine Metabolite 3,4-Dihydroxyphenylacetaldehyde in PC12 Cells. J. Pharmacol. Exp. Ther. 356 (2), 483–492. 10.1124/jpet.115.230201 26574516 PMC4746494

[B66] Goldstein D. S. Mezey E. Yamamoto T. Aneman A. Friberg P. Eisenhofer G. (1995). Is There a Third Peripheral Catecholaminergic System? Endogenous Dopamine as an Autocrine/paracrine Substance Derived from Plasma DOPA and Inactivated by Conjugation. Hypertens. Res. 18 (Suppl. 1), S93–S99. 10.1291/hypres.18.supplementi_s93 8529081

[B67] Goldstein D. S. (2020). The Catecholaldehyde Hypothesis: where MAO Fits in. J. Neural Transm. (Vienna). 127 (2), 169–177. 10.1007/s00702-019-02106-9 31807952 PMC10680281

[B68] Gordon S. Martinez F. O. (2010). Alternative Activation of Macrophages: Mechanism and Functions. Immunity. 32 (5), 593–604. 10.1016/j.immuni.2010.05.007 20510870

[B69] Graff L. A. Walker J. R. Bernstein C. N. (2009). Depression and Anxiety in Inflammatory Bowel Disease: a Review of Comorbidity and Management. Inflamm. Bowel Dis. 15 (7), 1105–1118. 10.1002/ibd.20873 19161177

[B70] Graves S. M. Xie Z. Stout K. A. Zampese E. Burbulla L. F. Shih J. C. (2020). Author Correction: Dopamine Metabolism by a Monoamine Oxidase Mitochondrial Shuttle Activates the Electron Transport Chain. Nat. Neurosci. 23 (2), 293. 10.1038/s41593-019-0583-0 PMC7333455 31911656

[B71] Grimsby J. Chen K. Wang L. J. Lan N. C. Shih J. C. (1991). Human Monoamine Oxidase A and B Genes Exhibit Identical Exon-Intron Organization. Proc. Natl. Acad. Sci. U S A. 88 (9), 3637–3641. 10.1073/pnas.88.9.3637 2023912 PMC51507

[B72] Gruber R. (2019). Osteoimmunology: Inflammatory Osteolysis and Regeneration of the Alveolar Bone. J. Clin. Periodontol. 46 (Suppl. 21), 52–69. 10.1111/jcpe.13056 30623453

[B73] Gurevich E. V. Gainetdinov R. R. Gurevich V. V. (2016). G Protein-Coupled Receptor Kinases as Regulators of Dopamine Receptor Functions. Pharmacol. Res. 111, 1–16. 10.1016/j.phrs.2016.05.010 27178731 PMC5079267

[B74] Hanami K. Nakano K. Saito K. Okada Y. Yamaoka K. Kubo S. (2013a). Dopamine D2-like Receptor Signaling Suppresses Human Osteoclastogenesis. Bone. 56 (1), 1–8. 10.1016/j.bone.2013.04.019 23631878

[B75] Hanami K. Nakano K. Tanaka Y. (2013b). [Dopamine Receptor Signaling Regulates Human Osteoclastogenesis]. Nihon Rinsho Meneki Gakkai Kaishi. 36 (1), 35–39. 10.2177/jsci.36.35 23445730

[B76] Handa K. Kiyohara S. Yamakawa T. Ishikawa K. Hosonuma M. Sakai N. (2019). Bone Loss Caused by Dopaminergic Degeneration and Levodopa Treatment in Parkinson's Disease Model Mice. Sci. Rep. 9 (1), 13768. 10.1038/s41598-019-50336-4 31551490 PMC6760231

[B77] Harmony J. A. Himes R. H. Schowen R. L. (1975). The Monovalent Cation-Induced Association of Formyltetrahydrofolate Synthetase Subunits: a Solvent Isotope Effect. Biochemistry 14 (24), 5379–5386. 10.1021/bi00695a023 1191644

[B78] Hasan F. McCrodden J. M. Kennedy N. P. Tipton K. F. (1988). The Involvement of Intestinal Monoamine Oxidase in the Transport and Metabolism of Tyramine. J. Neural Transm. Suppl. 26, 1–9. 3162948

[B79] Hunter C. A. Jones S. A. (2015). IL-6 as a Keystone Cytokine in Health and Disease. Nat. Immunol. 16 (5), 448–457. 10.1038/ni.3153 25898198

[B80] Iacovino L. G. Magnani F. Binda C. (2018). The Structure of Monoamine Oxidases: Past, Present, and Future. J. Neural Transm. (Vienna). 125 (11), 1567–1579. 10.1007/s00702-018-1915-z 30167931

[B81] Ise Y. Yamaguchi K. Sato K. Yamamura Y. Kitamura F. Tamatani T. (1988). Molecular Mechanisms Underlying Lymphocyte Recirculation. I. Functional, Phenotypical and Morphological Characterization of High Endothelial Cells Cultured In Vitro. Eur. J. Immunol. 18 (8), 1235–1244. 10.1002/eji.1830180814 3046950

[B82] Jage J. (1989). [Methadone--pharmacokinetics and Pharmacodynamics of an Opiate]. Anaesthesist. 38 (4), 159–166. 2567128

[B83] Jenei-Lanzl Z. Capellino S. Kees F. Fleck M. Lowin T. Straub R. H. (2015). Anti-inflammatory Effects of Cell-Based Therapy with Tyrosine Hydroxylase-Positive Catecholaminergic Cells in Experimental Arthritis. Ann. Rheum. Dis. 74 (2), 444–451. 10.1136/annrheumdis-2013-203925 24297380

[B84] Jiang J. L. Qiu Y. H. Peng Y. P. Wang J. J. (2006). Immunoregulatory Role of Endogenous Catecholamines Synthesized by Immune Cells. Sheng Li Xue Bao. 58 (4), 309–317. 16906330

[B85] Jinsmaa Y. Florang V. R. Rees J. N. Anderson D. G. Strack S. Doorn J. A. (2009). Products of Oxidative Stress Inhibit Aldehyde Oxidation and Reduction Pathways in Dopamine Catabolism Yielding Elevated Levels of a Reactive Intermediate. Chem. Res. Toxicol. 22 (5), 835–841. 10.1021/tx800405v 19388687 PMC2696154

[B86] Joshi A. U. Van Wassenhove L. D. Logas K. R. Minhas P. S. Andreasson K. I. Weinberg K. I. (2019). Aldehyde Dehydrogenase 2 Activity and Aldehydic Load Contribute to Neuroinflammation and Alzheimer's Disease Related Pathology. Acta Neuropathol. Commun. 7 (1), 190. 10.1186/s40478-019-0839-7 31829281 PMC6907112

[B87] Jung-Klawitter S. Kuseyri Hubschmann O. (2019). Analysis of Catecholamines and Pterins in Inborn Errors of Monoamine Neurotransmitter Metabolism-From Past to Future. Cells. 8 (8). 10.3390/cells8080867 PMC6721669 31405045

[B88] Kalogeris T. Baines C. P. Krenz M. Korthuis R. J. (2016). Ischemia/Reperfusion. Compr. Physiol. 7 (1), 113–170. 10.1002/cphy.c160006 28135002 PMC5648017

[B89] Kaludercic N. Carpi A. Menabo R. Di Lisa F. Paolocci N. (2011). Monoamine Oxidases (MAO) in the Pathogenesis of Heart Failure and Ischemia/reperfusion Injury. Biochim. Biophys. Acta 1813 (7), 1323–1332. 10.1016/j.bbamcr.2010.09.010 20869994 PMC3030628

[B90] Kaludercic N. Mialet-Perez J. Paolocci N. Parini A. Di Lisa F. (2014). Monoamine Oxidases as Sources of Oxidants in the Heart. J. Mol. Cel Cardiol. 73, 34–42. 10.1016/j.yjmcc.2013.12.032 PMC4048760 24412580

[B91] Kast R. E. (1998). Crohn's Disease Remission with Phenelzine Treatment. Gastroenterology. 115 (4), 1034–1035. 10.1016/s0016-5085(98)70292-6 9786733

[B92] Kim Y. Seo J. H. Kim H. (2011). Beta-Carotene and Lutein Inhibit Hydrogen Peroxide-Induced Activation of NF-kappaB and IL-8 Expression in Gastric Epithelial AGS Cells. J. Nutr. Sci. Vitaminol (Tokyo). 57 (3), 216–223. 10.3177/jnsv.57.216 21908944

[B93] Klein M. O. Battagello D. S. Cardoso A. R. Hauser D. N. Bittencourt J. C. Correa R. G. (2019). Dopamine: Functions, Signaling, and Association with Neurological Diseases. Cell Mol Neurobiol. 39 (1), 31–59. 10.1007/s10571-018-0632-3 30446950 PMC11469830

[B94] Knoll J. Magyar K. (1972). Some Puzzling Pharmacological Effects of Monoamine Oxidase Inhibitors. Adv. Biochem. Psychopharmacol. 5, 393–408. 5066229

[B95] Koide M. Kobayashi Y. Yamashita T. Uehara S. Nakamura M. Hiraoka B. Y. (2017). Bone Formation Is Coupled to Resorption via Suppression of Sclerostin Expression by Osteoclasts. J. Bone Miner Res. 32 (10), 2074–2086. 10.1002/jbmr.3175 28543818

[B96] Kolmus K. Tavernier J. Gerlo S. (2015). beta2-Adrenergic Receptors in Immunity and Inflammation: Stressing NF-kappaB. Brain Behav. Immun. 45, 297–310. 10.1016/j.bbi.2014.10.007 25459102

[B97] Kunduzova O. R. Bianchi P. Parini A. Cambon C. (2002a). Hydrogen Peroxide Production by Monoamine Oxidase during Ischemia/reperfusion. Eur. J. Pharmacol. 448 (2-3), 225–230. 10.1016/s0014-2999(02)01913-1 12144945

[B98] Kunduzova O. R. Bianchi P. Pizzinat N. Escourrou G. Seguelas M. H. Parini A. (2002b). Regulation of JNK/ERK Activation, Cell Apoptosis, and Tissue Regeneration by Monoamine Oxidases after Renal Ischemia-Reperfusion. FASEB J. 16 (9), 1129–1131. 10.1096/fj.01-1008fje 12039844

[B99] Laengle U. W. Trendelenburg A. U. Markstein R. Nogues V. Provencher-Bollinger A. Roman D. (2006). GLC756 Decreases TNF-Alpha via an Alpha2 and Beta2 Adrenoceptor Related Mechanism. Exp. Eye Res. 83 (5), 1246–1251. 10.1016/j.exer.2006.07.001 16938291

[B100] Lesniak A. Aarnio M. Diwakarla S. Norberg T. Nyberg F. Gordh T. (2018). Characterization of the Binding Site for D-Deprenyl in Human Inflamed Synovial Membrane. Life Sci. 194, 26–33. 10.1016/j.lfs.2017.12.003 29221756

[B101] Lesniak A. Aarnio M. Jonsson A. Norberg T. Nyberg F. Gordh T. (2016). High-throughput Screening and Radioligand Binding Studies Reveal Monoamine Oxidase-B as the Primary Binding Target for D-Deprenyl. Life Sci. 152, 231–237. 10.1016/j.lfs.2016.03.058 27058977

[B102] Liao C. P. Lin T. P. Li P. C. Geary L. A. Chen K. Vaikari V. P. (2018). Loss of MAO-A in Epithelia Inhibits Adenocarcinoma Development, Cell Proliferation and Cancer Stem Cells in Prostate. Oncogene. 37 (38), 5175–5190. 10.1038/s41388-018-0325-x 29844571 PMC7500062

[B103] Lieb J. (1983). Remission of Rheumatoid Arthritis and Other Disorders of Immunity in Patients Taking Monoamine Oxidase Inhibitors. Int. J. Immunopharmacol 5 (4), 353–357. 10.1016/0192-0561(83)90039-5 6629596

[B104] Lin A. Song C. Kenis G. Bosmans E. De Jongh R. Scharpe S. (2000). The In Vitro Immunosuppressive Effects of Moclobemide in Healthy Volunteers. J. Affect Disord. 58 (1), 69–74. 10.1016/s0165-0327(99)00076-2 10760560

[B105] Linnman C. Appel L. Fredrikson M. Gordh T. Soderlund A. Langstrom B. (2011). Elevated [11C]-D-Deprenyl Uptake in Chronic Whiplash Associated Disorder Suggests Persistent Musculoskeletal Inflammation. PLoS One 6 (4), e19182. 10.1371/journal.pone.0019182 21541010 PMC3079741

[B106] Liu Y. Feng S. Subedi K. Wang H. (2020). Attenuation of Ischemic Stroke-Caused Brain Injury by a Monoamine Oxidase Inhibitor Involves Improved Proteostasis and Reduced Neuroinflammation. Mol. Neurobiol. 57 (2), 937–948. 10.1007/s12035-019-01788-2 31620993 PMC7035161

[B107] Liu Z. Yang K. Yan X. Wang T. Jiang T. Zhou Q. (2019). The Effects of Tranylcypromine on Osteoclastogenesis In Vitro and In Vivo. FASEB J. 33 (9), 9828–9841. 10.1096/fj.201802242RR 31291555

[B108] Lundborg P. (1969). Effect of Reserpine on the Subcellular Distribution of 3H-Alpha-Methylnoradrenaline in the Mouse Heart. Br. J. Pharmacol. 36 (2), 386–392. 10.1111/j.1476-5381.1969.tb09514.x 5787673 PMC1703387

[B109] Matt S. M. Gaskill P. J. (2020). Where Is Dopamine and How Do Immune Cells See it?: Dopamine-Mediated Immune Cell Function in Health and Disease. J. Neuroimmune Pharmacol. 15 (1), 114–164. 10.1007/s11481-019-09851-4 31077015 PMC6842680

[B110] Melnikov M. Rogovskii V. Boyksmall o C. A. Pashenkov M. (2020). Dopaminergic Therapeutics in Multiple Sclerosis: Focus on Th17-Cell Functions. J. Neuroimmune Pharmacol. 15 (1), 37–47. 10.1007/s11481-019-09852-3 31011885

[B111] Menazza S. Blaauw B. Tiepolo T. Toniolo L. Braghetta P. Spolaore B. (2010). Oxidative Stress by Monoamine Oxidases Is Causally Involved in Myofiber Damage in Muscular Dystrophy. Hum. Mol. Genet. 19 (21), 4207–4215. 10.1093/hmg/ddq339 20716577

[B112] Motyl K. J. Beauchemin M. Barlow D. Le P. T. Nagano K. Treyball A. (2017). A Novel Role for Dopamine Signaling in the Pathogenesis of Bone Loss from the Atypical Antipsychotic Drug Risperidone in Female Mice. Bone. 103, 168–176. 10.1016/j.bone.2017.07.008 28689816 PMC5573184

[B113] Najafi M. Noroozi E. Javadi A. Badalzadeh R. (2018). Anti-arrhythmogenic and Anti-inflammatory Effects of Troxerutin in Ischemia/reperfusion Injury of Diabetic Myocardium. Biomed. Pharmacother. 102, 385–391. 10.1016/j.biopha.2018.03.047 29573617

[B114] Nakashioya H. Nakano K. Watanabe N. Miyasaka N. Matsushita S. Kohsaka H. (2011). Therapeutic Effect of D1-like Dopamine Receptor Antagonist on Collagen-Induced Arthritis of Mice. Mod. Rheumatol. 21 (3), 260–266. 10.1007/s10165-010-0387-2 21188452

[B115] Nelson M. M. Baba S. P. Anderson E. J. (2017). Biogenic Aldehydes as Therapeutic Targets for Cardiovascular Disease. Curr. Opin. Pharmacol. 33, 56–63. 10.1016/j.coph.2017.04.004 28528297 PMC5563970

[B183] Nolan R. Gaskill P. J. (2019). The Role of Catecholamines in HIV Neuropathogenesis. Brain Res. 1702, 54–73. 10.1016/j.brainres.2018.04.030 29705605 PMC6204123

[B118] Opal S. M. DePalo V. A. (2000). Anti-inflammatory Cytokines. Chest. 117 (4), 1162–1172. 10.1378/chest.117.4.1162 10767254

[B119] Oyewole A. O. Birch-Machin M. A. (2015). Mitochondria-targeted Antioxidants. FASEB J. 29 (12), 4766–4771. 10.1096/fj.15-275404 26253366

[B120] Palomino D. C. Marti L. C. (2015). Chemokines and Immunity. Einstein (Sao Paulo). 13 (3), 469–473. 10.1590/S1679-45082015RB3438 26466066 PMC4943798

[B121] Pan C. Xing J. H. Zhang C. Zhang Y. M. Zhang L. T. Wei S. J. (2016). Aldehyde Dehydrogenase 2 Inhibits Inflammatory Response and Regulates Atherosclerotic Plaque. Oncotarget. 7 (24), 35562–35576. 10.18632/oncotarget.9384 27191745 PMC5094945

[B122] Panarsky R. Luques L. Weinstock M. (2012). Anti-inflammatory Effects of Ladostigil and its Metabolites in Aged Rat Brain and in Microglial Cells. J. Neuroimmune Pharmacol. 7 (2), 488–498. 10.1007/s11481-012-9358-z 22454040

[B123] Pavlin M. Repic M. Vianello R. Mavri J. (2016). The Chemistry of Neurodegeneration: Kinetic Data and Their Implications. Mol. Neurobiol. 53 (5), 3400–3415. 10.1007/s12035-015-9284-1 26081152

[B124] Peehl D. M. Coram M. Khine H. Reese S. Nolley R. Zhao H. (2008). The Significance of Monoamine Oxidase-A Expression in High Grade Prostate Cancer. J. Urol. 180 (5), 2206–2211. 10.1016/j.juro.2008.07.019 18804811 PMC2743604

[B125] Pinoli M. Marino F. Cosentino M. (2017). Dopaminergic Regulation of Innate Immunity: a Review. J. Neuroimmune Pharmacol. 12 (4), 602–623. 10.1007/s11481-017-9749-2 28578466

[B126] Pizzinat N. Copin N. Vindis C. Parini A. Cambon C. (1999). Reactive Oxygen Species Production by Monoamine Oxidases in Intact Cells. Naunyn Schmiedebergs Arch. Pharmacol. 359 (5), 428–431. 10.1007/pl00005371 10498294

[B127] Pletscher A. (1991). The Discovery of Antidepressants: a Winding Path. Experientia. 47 (1), 4–8. 10.1007/BF02041242 1999242

[B128] Prah A. Purg M. Stare J. Vianello R. Mavri J. (2020). How Monoamine Oxidase A Decomposes Serotonin: An Empirical Valence Bond Simulation of the Reactive Step. J. Phys. Chem. B. 124 (38), 8259–8265. 10.1021/acs.jpcb.0c06502 32845149 PMC7520887

[B129] Pravda J. (2020). Hydrogen Peroxide and Disease: towards a Unified System of Pathogenesis and Therapeutics. Mol. Med. 26 (1), 41. 10.1186/s10020-020-00165-3 32380940 PMC7204068

[B130] Qiu Y. H. Cheng C. Dai L. Peng Y. P. (2005). Effect of Endogenous Catecholamines in Lymphocytes on Lymphocyte Function. J. Neuroimmunol. 167 (1-2), 45–52. 10.1016/j.jneuroim.2005.06.007 15996757

[B131] Ramsay R. R. Albreht A. (2018). Kinetics, Mechanism, and Inhibition of Monoamine Oxidase. J. Neural Transm. (Vienna) 125 (11), 1659–1683. 10.1007/s00702-018-1861-9 29516165

[B132] Ratiu C. Utu D. Petrus A. Norbert P. Olariu S. Duicu O. (2018). Monoamine Oxidase Inhibition Improves Vascular Function and Reduces Oxidative Stress in Rats with Lipopolysaccharide-Induced Inflammation. Gen. Physiol. Biophys. 37 (6), 687–694. 10.4149/gpb_2018014 30061472

[B133] Rea I. M. Gibson D. S. McGilligan V. McNerlan S. E. Alexander H. D. Ross O. A. (2018). Age and Age-Related Diseases: Role of Inflammation Triggers and Cytokines. Front. Immunol. 9, 586. 10.3389/fimmu.2018.00586 29686666 PMC5900450

[B134] Redlich K. Smolen J. S. (2012). Inflammatory Bone Loss: Pathogenesis and Therapeutic Intervention. Nat. Rev. Drug Discov. 11 (3), 234–250. 10.1038/nrd3669 22378270

[B135] Rees J. N. Florang V. R. Eckert L. L. Doorn J. A. (2009). Protein Reactivity of 3,4-dihydroxyphenylacetaldehyde, a Toxic Dopamine Metabolite, Is Dependent on Both the Aldehyde and the Catechol. Chem. Res. Toxicol. 22 (7), 1256–1263. 10.1021/tx9000557 19537779 PMC2717024

[B136] Repic M. Vianello R. Purg M. Duarte F. Bauer P. Kamerlin S. C. (2014). Empirical Valence Bond Simulations of the Hydride Transfer Step in the Monoamine Oxidase B Catalyzed Metabolism of Dopamine. Proteins. 82 (12), 3347–3355. 10.1002/prot.24690 25220264

[B137] Robinson J. B. (1985). Stereoselectivity and Isoenzyme Selectivity of Monoamine Oxidase Inhibitors. Enantiomers of Amphetamine, N-Methylamphetamine and Deprenyl. Biochem. Pharmacol. 34 (23), 4105–4108. 10.1016/0006-2952(85)90201-1 3933519

[B138] Rodriguez M. J. Saura J. Billett E. E. Finch C. C. Mahy N. (2001). Cellular Localization of Monoamine Oxidase A and B in Human Tissues outside of the Central Nervous System. Cell Tissue Res. 304 (2), 215–220. 10.1007/s004410100361 11396715

[B139] Rodriguez-Munoz A. Garcia-Garcia G. Menor F. Millan J. M. Tomas-Vila M. Jaijo T. (2018). The Importance of Biochemical and Genetic Findings in the Diagnosis of Atypical Norrie Disease. Clin. Chem. Lab. Med. 56 (2), 229–235. 10.1515/cclm-2017-0226 28742514

[B140] Sanchez-Rodriguez R. Munari F. Angioni R. Venegas F. Agnellini A. Castro-Gil M. P. (2020). Targeting Monoamine Oxidase to Dampen NLRP3 Inflammasome Activation in Inflammation. Cell Mol Immunol. 10.1038/s41423-020-0441-8 PMC8093264 32346102

[B141] Sapkota M. Wyatt T. A. (2015). Alcohol, Aldehydes, Adducts and Airways. Biomolecules 5 (4), 2987–3008. 10.3390/biom5042987 26556381 PMC4693266

[B142] Sawada M. Imamura K. Nagatsu T. (2006). Role of Cytokines in Inflammatory Process in Parkinson's Disease. J. Neural Transm. Suppl (70), 373–381. 10.1007/978-3-211-45295-0_57 17017556

[B143] Scanzano A. Cosentino M. (2015). Adrenergic Regulation of Innate Immunity: a Review. Front. Pharmacol. 6, 171. 10.3389/fphar.2015.00171 26321956 PMC4534859

[B144] Scanzano A. Schembri L. Rasini E. Luini A. Dallatorre J. Legnaro M. (2015). Adrenergic Modulation of Migration, CD11b and CD18 Expression, ROS and Interleukin-8 Production by Human Polymorphonuclear Leukocytes. Inflamm. Res. 64 (2), 127–135. 10.1007/s00011-014-0791-8 25561369

[B145] Scott D. L. Wolfe F. Huizinga T. W. (2010). Rheumatoid Arthritis. Lancet. 376 (9746), 1094–1108. 10.1016/S0140-6736(10)60826-4 20870100

[B146] Shih J. C. (2018). Monoamine Oxidase Isoenzymes: Genes, Functions and Targets for Behavior and Cancer Therapy. J. Neural Transm. (Vienna). 125 (11), 1553–1566. 10.1007/s00702-018-1927-8 30259128 PMC6245662

[B147] Sivasubramaniam S. D. Finch C. C. Billett M. A. Baker P. N. Billett E. E. (2002). Monoamine Oxidase Expression and Activity in Human Placentae from Pre-eclamptic and Normotensive Pregnancies. Placenta. 23 (2-3), 163–171. 10.1053/plac.2001.0770 11945082

[B148] Sivasubramaniam S. D. Finch C. C. Rodriguez M. J. Mahy N. Billett E. E. (2003). A Comparative Study of the Expression of Monoamine Oxidase-A and -B mRNA and Protein in Non-CNS Human Tissues. Cel Tissue Res. 313 (3), 291–300. 10.1007/s00441-003-0765-6 12898212

[B149] Smolen J. S. Aletaha D. McInnes I. B. (2016). Rheumatoid Arthritis. Lancet. 388 (10055), 2023–2038. 10.1016/S0140-6736(16)30173-8 27156434

[B150] Snow W. M. Albensi B. C. (2016). Neuronal Gene Targets of NF-kappaB and Their Dysregulation in Alzheimer's Disease. Front. Mol. Neurosci. 9, 118. 10.3389/fnmol.2016.00118 27881951 PMC5101203

[B151] Son S. Y. Ma J. Kondou Y. Yoshimura M. Yamashita E. Tsukihara T. (2008). Structure of Human Monoamine Oxidase A at 2.2-A Resolution: the Control of Opening the Entry for Substrates/inhibitors. Proc. Natl. Acad. Sci. U S A. 105 (15), 5739–5744. 10.1073/pnas.0710626105 18391214 PMC2311356

[B152] Spengler R. N. Chensue S. W. Giacherio D. A. Blenk N. Kunkel S. L. (1994). Endogenous Norepinephrine Regulates Tumor Necrosis Factor-Alpha Production from Macrophages In Vitro. J. Immunol. 152 (6), 3024–3031. 8144901

[B153] Straub R. H. Schradin C. (2016). Chronic Inflammatory Systemic Diseases: An Evolutionary Trade-Off between Acutely Beneficial but Chronically Harmful Programs. Evol. Med. Public Health 2016 (1), 37–51. 10.1093/emph/eow001 26817483 PMC4753361

[B154] Strosberg A. D. (1993). Structure, Function, and Regulation of Adrenergic Receptors. Protein Sci. 2 (8), 1198–1209. 10.1002/pro.5560020802 8401205 PMC2142449

[B155] Strowig T. Henao-Mejia J. Elinav E. Flavell R. (2012). Inflammasomes in Health and Disease. Nature 481 (7381), 278–286. 10.1038/nature10759 22258606

[B156] Sturza A. Popoiu C. M. Ionica M. Duicu O. M. Olariu S. Muntean D. M. (2019). Monoamine Oxidase-Related Vascular Oxidative Stress in Diseases Associated with Inflammatory Burden. Oxid Med. Cel Longev. 2019, 8954201. 10.1155/2019/8954201 PMC6501417 31178977

[B157] Subhramanyam C. S. Wang C. Hu Q. Dheen S. T. (2019). Microglia-mediated Neuroinflammation in Neurodegenerative Diseases. Semin. Cel Dev Biol. 94, 112–120. 10.1016/j.semcdb.2019.05.004 31077796

[B158] Sulzer D. Zecca L. (2000). Intraneuronal Dopamine-Quinone Synthesis: a Review. Neurotox Res. 1 (3), 181–195. 10.1007/BF03033289 12835101

[B159] Tanaka T. Narazaki M. Kishimoto T. (2014). IL-6 in Inflammation, Immunity, and Disease. Cold Spring Harb Perspect. Biol. 6 (10), a016295. 10.1101/cshperspect.a016295 25190079 PMC4176007

[B160] Tandaric T. Prah A. Stare J. Mavri J. Vianello R. (2020). Hydride Abstraction as the Rate-Limiting Step of the Irreversible Inhibition of Monoamine Oxidase B by Rasagiline and Selegiline: A Computational Empirical Valence Bond Study. Int. J. Mol. Sci. 21 (17). 10.3390/ijms21176151 PMC7503497 32858935

[B161] Teitelbaum S. L. Ross F. P. (2003). Genetic Regulation of Osteoclast Development and Function. Nat. Rev. Genet. 4 (8), 638–649. 10.1038/nrg1122 12897775

[B162] Thomas Broome S. Louangaphay K. Keay K. A. Leggio G. M. Musumeci G. Castorina A. (2020). Dopamine: an Immune Transmitter. Neural Regen. Res. 15 (12), 2173–2185. 10.4103/1673-5374.284976 32594028 PMC7749467

[B163] Thorpe L. W. Westlund K. N. Kochersperger L. M. Abell C. W. Denney R. M. (1987). Immunocytochemical Localization of Monoamine Oxidases A and B in Human Peripheral Tissues and Brain. J. Histochem. Cytochem. 35 (1), 23–32. 10.1177/35.1.3025289 3025289

[B164] Tipton K. F. (2018). 90 Years of Monoamine Oxidase: Some Progress and Some Confusion. J. Neural Transm. (Vienna). 125 (11), 1519–1551. 10.1007/s00702-018-1881-5 29637260

[B165] Tisoncik J. R. Korth M. J. Simmons C. P. Farrar J. Martin T. R. Katze M. G. (2012). Into the Eye of the Cytokine Storm. Microbiol. Mol. Biol. Rev. 76 (1), 16–32. 10.1128/MMBR.05015-11 22390970 PMC3294426

[B166] Tomaz V. S. Chaves Filho A. J. M. Cordeiro R. C. Juca P. M. Soares M. V. R. Barroso P. N. (2020). Antidepressants of Different Classes Cause Distinct Behavioral and Brain Pro- and Anti-inflammatory Changes in Mice Submitted to an Inflammatory Model of Depression. J. Affect Disord. 268, 188–200. 10.1016/j.jad.2020.03.022 32174477

[B167] Tranah T. H. Vijay G. K. Ryan J. M. Shawcross D. L. (2013). Systemic Inflammation and Ammonia in Hepatic Encephalopathy. Metab. Brain Dis. 28 (1), 1–5. 10.1007/s11011-012-9370-2 23224356

[B168] Tripathi A. C. Upadhyay S. Paliwal S. Saraf S. K. (2018). Privileged Scaffolds as MAO Inhibitors: Retrospect and Prospects. Eur. J. Med. Chem. 145, 445–497. 10.1016/j.ejmech.2018.01.003 29335210

[B169] Trudler D. Weinreb O. Mandel S. A. Youdim M. B. Frenkel D. (2014). DJ-1 Deficiency Triggers Microglia Sensitivity to Dopamine toward a Pro-inflammatory Phenotype that Is Attenuated by Rasagiline. J. Neurochem. 129 (3), 434–447. 10.1111/jnc.12633 24355073

[B170] van der Vliet A. Janssen-Heininger Y. M. (2014). Hydrogen Peroxide as a Damage Signal in Tissue Injury and Inflammation: Murderer, Mediator, or Messenger? J. Cel Biochem. 115 (3), 427–435. 10.1002/jcb.24683 PMC4363740 24122865

[B171] Vitiello L. Marabita M. Sorato E. Nogara L. Forestan G. Mouly V. (2018). Drug Repurposing for Duchenne Muscular Dystrophy: The Monoamine Oxidase B Inhibitor Safinamide Ameliorates the Pathological Phenotype in Mdx Mice and in Myogenic Cultures from DMD Patients. Front. Physiol. 9, 1087. 10.3389/fphys.2018.01087 30154729 PMC6102489

[B172] Wang J. Edmondson D. E. (2011). Topological Probes of Monoamine Oxidases A and B in Rat Liver Mitochondria: Inhibition by TEMPO-Substituted Pargyline Analogues and Inactivation by Proteolysis. Biochemistry 50 (13), 2499–2505. 10.1021/bi101722b 21341713 PMC3068223

[B173] Wang L. Han L. Xue P. Hu X. Wong S. W. Deng M. (2021). Dopamine Suppresses Osteoclast Differentiation via cAMP/PKA/CREB Pathway. Cell Signal 78, 109847. 10.1016/j.cellsig.2020.109847 33242564 PMC8691485

[B174] Winterbourn C. C. (2013). The Biological Chemistry of Hydrogen Peroxide. Methods Enzymol. 528, 3–25. 10.1016/B978-0-12-405881-1.00001-X 23849856

[B175] Wittmann C. Chockley P. Singh S. K. Pase L. Lieschke G. J. Grabher C. (2012). Hydrogen Peroxide in Inflammation: Messenger, Guide, and Assassin. Adv. Hematol. 2012, 541471. 10.1155/2012/541471 22737171 PMC3378963

[B176] Wu J. B. Shao C. Li X. Li Q. Hu P. Shi C. (2014). Monoamine Oxidase A Mediates Prostate Tumorigenesis and Cancer Metastasis. J. Clin. Invest. 124 (7), 2891–2908. 10.1172/JCI70982 24865426 PMC4071401

[B177] Wu J. B. Yin L. Shi C. Li Q. Duan P. Huang J. M. (2017). MAO-A-dependent Activation of Shh-IL6-RANKL Signaling Network Promotes Prostate *Cancer* Metastasis by Engaging Tumor-Stromal Cell Interactions. Cancer Cell 31 (3), 368–382. 10.1016/j.ccell.2017.02.003 28292438

[B178] Xiong J. Piemontese M. Onal M. Campbell J. Goellner J. J. Dusevich V. (2015). Osteocytes, Not Osteoblasts or Lining Cells, Are the Main Source of the RANKL Required for Osteoclast Formation in Remodeling Bone. PLoS One 10 (9), e0138189. 10.1371/journal.pone.0138189 26393791 PMC4578942

[B179] Yang H. Xu Y. Zhu M. Gu Y. Zhang W. Shao H. (2016). Inhibition of Titanium-Particle-Induced Inflammatory Osteolysis after Local Administration of Dopamine and Suppression of Osteoclastogenesis via D2-like Receptor Signaling Pathway. Biomaterials 80, 1–10. 10.1016/j.biomaterials.2015.11.046 26695376

[B180] Yin L. Li J. Liao C. P. Jason Wu B. (2018). Monoamine Oxidase Deficiency Causes Prostate Atrophy and Reduces Prostate Progenitor Cell Activity. Stem Cells. 36 (8), 1249–1258. 10.1002/stem.2831 29637670

[B181] Yoshioka Y. Sugino Y. Shibagaki F. Yamamuro A. Ishimaru Y. Maeda S. (2020). Dopamine Attenuates Lipopolysaccharide-Induced Expression of Proinflammatory Cytokines by Inhibiting the Nuclear Translocation of NF-kappaB P65 through the Formation of Dopamine Quinone in Microglia. Eur. J. Pharmacol. 866, 172826. 10.1016/j.ejphar.2019.172826 31790652

[B182] Youdim M. B. Edmondson D. Tipton K. F. (2006). The Therapeutic Potential of Monoamine Oxidase Inhibitors. Nat. Rev. Neurosci. 7 (4), 295–309. 10.1038/nrn1883 16552415

